# PRR11 promotes ccRCC tumorigenesis by regulating E2F1 stability

**DOI:** 10.1172/jci.insight.145172

**Published:** 2021-10-08

**Authors:** Siming Chen, Zhiwen He, Tianchen Peng, Fenfang Zhou, Gang Wang, Kaiyu Qian, Lingao Ju, Yu Xiao, Xinghuan Wang

**Affiliations:** 1 Department of Urology and; 2 Department of Biological Repositories, Zhongnan Hospital of Wuhan University, Wuhan, China.; 3 Human Genetic Resource Preservation Center of Hubei Province, Wuhan, China.; 4 Research Center of Wuhan for Infectious Diseases and Cancer, Chinese Academy of Medical Sciences, Wuhan, China.; 5 Laboratory of Precision Medicine, Zhongnan Hospital of Wuhan University, Wuhan, China.; 6 Medical Research Institute, Wuhan University, Wuhan, China.

**Keywords:** Cell Biology, Nephrology, Cancer, Cell cycle

## Abstract

Proline rich 11 (PRR11), a novel tumor-related gene, has been identified in different tumors. However, the relevant biological functions of PRR11 in human clear cell renal cell carcinoma (ccRCC) have not been studied. In this study, we first identified PRR11 as a biomarker of ccRCC and predictor of poor prognosis by bioinformatics. Then, we showed that *PRR11* silencing substantially reduced ccRCC cell proliferation and migration in vitro and in vivo. Importantly, we found that PRR11 induced the degradation of the E2F1 protein through its interaction with E2F1, and PRR11 reduced the stability of the E2F1 protein in ccRCC cells, thereby affecting cell cycle progression. Further results indicated that the downregulation of *E2F1* expression partially reversed the changes in ccRCC cell biology caused by PRR11 deletion. In addition, we showed that PRR11 was a target gene of c-Myc. The transcription factor c-Myc may have promoted the expression of PRR11 in ccRCC cells by binding to the PRR11 promoter region, thereby accelerating the progression of ccRCC. In summary, we found that PRR11 served as an oncogene in ccRCC, and PRR11 reduced the protein stability of E2F1 and could be activated by c-Myc.

## Graphical abstract

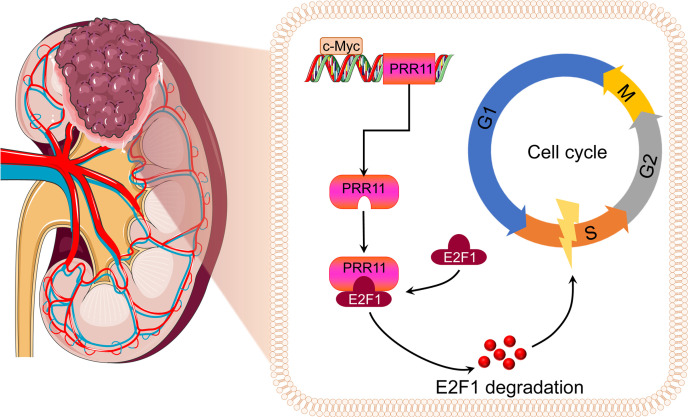

## Introduction

Renal cell carcinoma (RCC), a tumor associated with high morbidity and mortality, seriously endangers the lives and health of people worldwide. According to statistics, there were approximately 66,800 new cases and 23,400 deaths related to kidney cancer in China in 2015 (1). Clear cell RCC (ccRCC) accounts for the majority of RCC cases, and biallelic deactivation of the von Hippel-Lindau (*VHL*) gene by the deletion of chromosome 3p or mutation of the gene is a significant feature of ccRCC (2–4). The insensitivity of ccRCC to chemoradiotherapy and its susceptibility to recurrence affect the prognosis of ccRCC patients (5, 6). Therefore, an in-depth understanding of the biological mechanism underlying ccRCC will help in the development of new diagnostic and therapeutic methods.

Proline rich 11 (PRR11) is a protein composed of 360 amino acids, and its expression is widely upregulated in many tumors (7–11). Recent mechanistic studies showed that PRR11 promoted lung cancer development by regulating the expression of cell cycle–related genes (7). Other studies have indicated that *PRR11* knockdown could inhibit tumor occurrence by inducing autophagy in lung cancer cells (12). PRR11 has 2 conserved domains. One domain is the zinc finger domain, which can bind to DNA to regulate transcription, and the other domain is the proline-rich domain, which can bind to other domains and mediate protein-protein interactions, thus affecting the occurrence of tumors (13–17). Consistent with these results of functional domain analysis, we found that PRR11 could interact with E2F1 and reduce its protein stability, affecting the occurrence and development of ccRCC tumors.

As a controversial transcription factor, E2F1 not only promotes the proliferation of tumor cells but also induces senescence and apoptosis (18, 19). Overexpression of E2F1 can promote cell proliferation and tumor development, but deletion of E2F1 in mice leads to tumorigenesis, suggesting that E2F1 also plays a vital role in tumor inhibition (20). It was also confirmed that E2F1 could inhibit skin cancer development through ARF-p53–related pathways (21). Similar to other tumors, E2F1 is highly expressed in ccRCC (22, 23). However, Mans et al. found that increased expression of E2F1 could inhibit tumor growth by promoting cell senescence in RCC. Other studies demonstrated that increased E2F1 expression was significantly associated with favorable prognosis and tumor stage (24). Recent studies showed that Cyclin F controlled cell cycle progression via the proteasomal degradation of E2F1 in the late S and G2 phases, whereas the accumulation of E2F1 led to DNA damage and decreased cell viability (25). Interestingly, silencing *PRR11* expression increased the proportion of cells in the S phase, inhibiting cell viability and tumorigenic potential (7). Therefore, we hypothesized that PRR11 might cooperate with E2F1 to jointly regulate the progression of the cell cycle.

The expression of c-Myc is generally upregulated in different tumors (26–28). The overexpression of c-Myc has also been also confirmed in ccRCC tissues and cell lines (29). In addition, quantitative real-time PCR (qRT-PCR) analysis of clinical samples that we collected also confirmed that *c-Myc* expression was upregulated in ccRCC tissues. As a well-known transcription factor, c-Myc plays a crucial role in biological activities, such as cell cycle progression, apoptosis, and metabolism (30). Through bioinformatics analysis and transcription factor prediction, we hypothesized that PRR11 expression might be directly regulated by c-Myc.

## Results

### Bioinformatics analyses of PRR11 expression in The Cancer Genome Atlas.

We analyzed the expression level of PRR11 using the TIMER database and found that *PRR11* was expressed at higher levels in most human tumor tissues than in the corresponding normal tissues (Supplemental Figure 1A; supplemental material available online with this article; https://doi.org/10.1172/jci.insight.145172DS1). Based on The Cancer Genome Atlas (TCGA) database (539 ccRCC tissues and 72 normal kidney tissues), we showed that *PRR11* was highly expressed in patients with ccRCC (Supplemental Figure 1, B and C). In addition, survival analysis showed that patients with higher *PRR11* expression had shorter survival times (Supplemental Figure 1D). Notably, the expression of *PRR11* in ccRCC was significantly positively correlated with the grade and stage of ccRCC (Supplemental Figure 1, E and F). To further validate the diagnostic value of PRR11 expression, univariate and multivariate Cox analyses were performed, which demonstrated that high *PRR11* expression was closely associated with poor prognosis (Supplemental Table 1). According to the Cox analysis, a nomogram for prognostic assessment was constructed based on the additive assessment of the risk factors for ccRCC. The nomogram consists of upper and lower components. The upper section includes the scale (“Points”) that is used to calculate the weight of each variable (age, sex, grade, stage, T, M, N, and PRR11 expression). The lower section was then used to calculate the aggregate (“Total Points”) and the 1-, 3-, and 5-year survival rates of ccRCC patients (Supplemental Figure 2A). The nomogram could accurately calculate the 1-, 3-, and 5-year survival rates of ccRCC patients. The calibration curves showed that the predicted probability of the nomogram was consistent with the actual survival rate (Supplemental Figure 2, B–D). In addition, the 1-, 3-, and 5-year AUCs of the nomogram were 0.897 (95% CI, 0.848–0.947), 0.838 (95% CI, 0.775–0.901), and 0.793 (95% CI, 0.711–0.874), respectively, demonstrating very good predictive performance (Supplemental Figure 2, E–G). Therefore, we believe that PRR11 may be used as a biomarker to evaluate ccRCC progression in future studies.

### PRR11 is highly expressed in ccRCC and predicts a poor prognosis.

**Figure 1:**
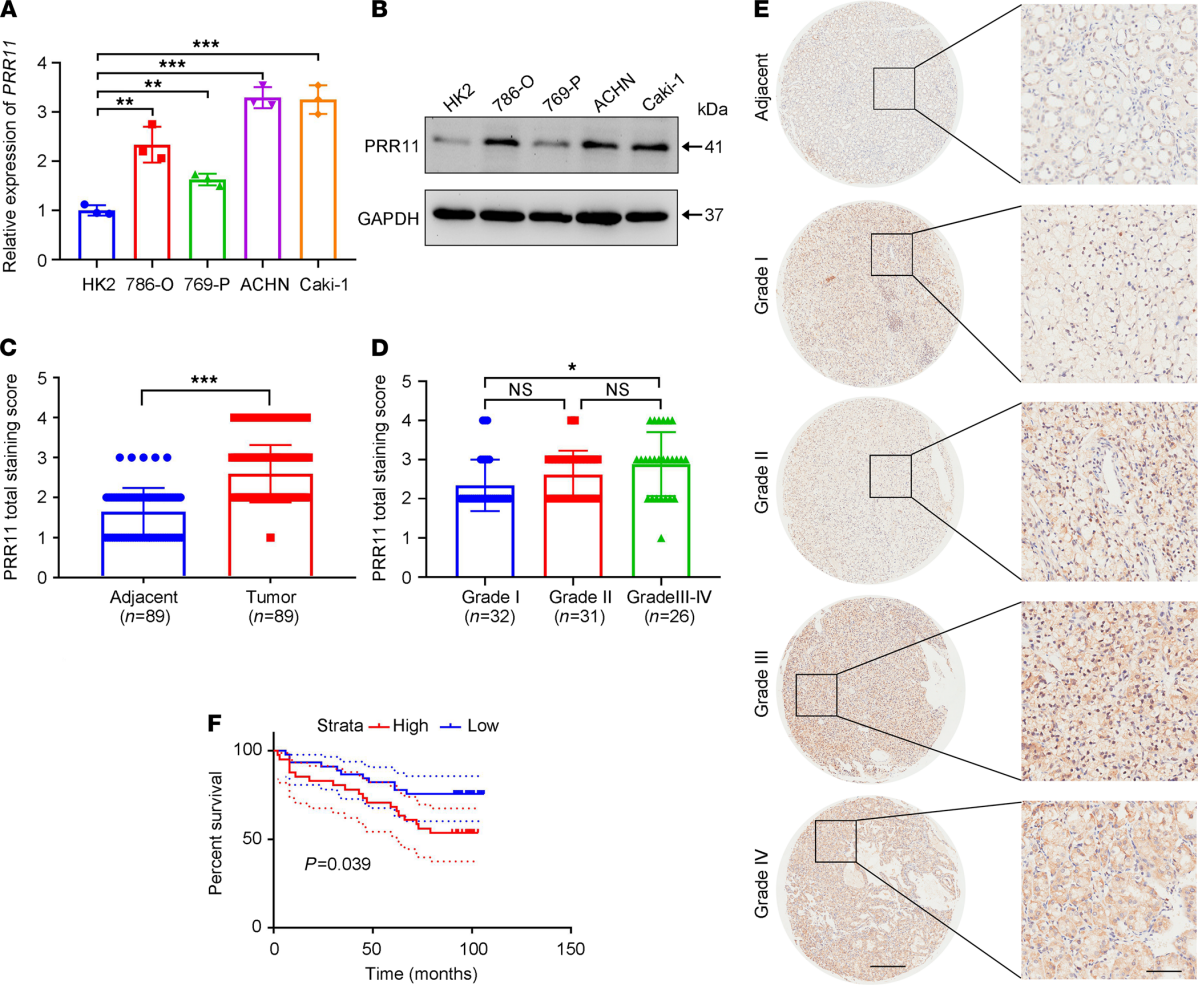
PRR11 is an independent factor of ccRCC. (**A** and **B**) The expression level of PRR11 in ccRCC cell lines (ACHN, 796-P, 786-O, and Caki-1 cells) and a human renal tubular epithelial cell line (HK2; *n* = 3). (**C**) PRR11 staining score of ccRCC tissues and adjacent tissues based on a tissue microarray (*n* = 89 ccRCC tissues and *n* = 89 adjacent tissues). (**D**) Correlation between the PRR11 staining score and the ccRCC pathological grade based on a tissue microarray (*n* = 32 for grade I, *n* = 31 for grade II, *n* = 25 for grade III, and *n* = 1 for grade IV). (**E**) Representative pattern of PRR11 protein expression in ccRCC tissues and adjacent tissues using tissue microarray sections. (**F**) Survival analysis of PRR11 expression based on a ccRCC tissue microarray. Scale bar: 400 μm (left) and 100 μm (right). The data are shown as mean ± SD. One-way ANOVA with Dunnett’s multiple comparisons test (**A**), 2-tailed *t* test (**C**), and 1-way ANOVA with Tukey’s multiple comparisons test (**D**) analyses were performed. *P < 0.05, **P < 0.01, ***P < 0.001. PRR11, proline rich 11; ccRCC, clear cell renal cell carcinoma.

Next, we investigated the expression of PRR11 in different ccRCC cell lines. Compared with that in HK2 cells, PRR11 expression in ccRCC cells was markedly increased at the mRNA and protein levels (Figure 1, A and B). IHC staining of the samples collected in our hospital showed that ccRCC tissues had higher PRR11 expression than normal adjacent kidney tissues. In addition, with the increase in ccRCC pathological grade, PRR11 expression also significantly increased (Supplemental Figure 3).

To further verify our hypothesis, IHC staining was used to detect PRR11 expression in tissue microarrays. According to the IHC results, PRR11 expression was significantly higher in ccRCC tissues than in normal tissues (Figure 1C). Moreover, compared with low-grade patients, high-grade patients had higher expression of PRR11 in their malignant tissues (Figure 1, D and E). Patients with ccRCC were divided into 2 groups based on the median IHC score, and survival was significantly lower in the high PRR11 expression group than in the low PRR11 expression group (Figure 1F). The clinical information about the tissue microarray is provided in Supplemental Figure 4. In summary, PRR11 expression was closely related to ccRCC progression and could have served as a marker for patients with ccRCC.

### Reduction in PRR11 expression inhibits ccRCC cell proliferation and increases the proportion of cells in the S phase.

**Figure 2:**
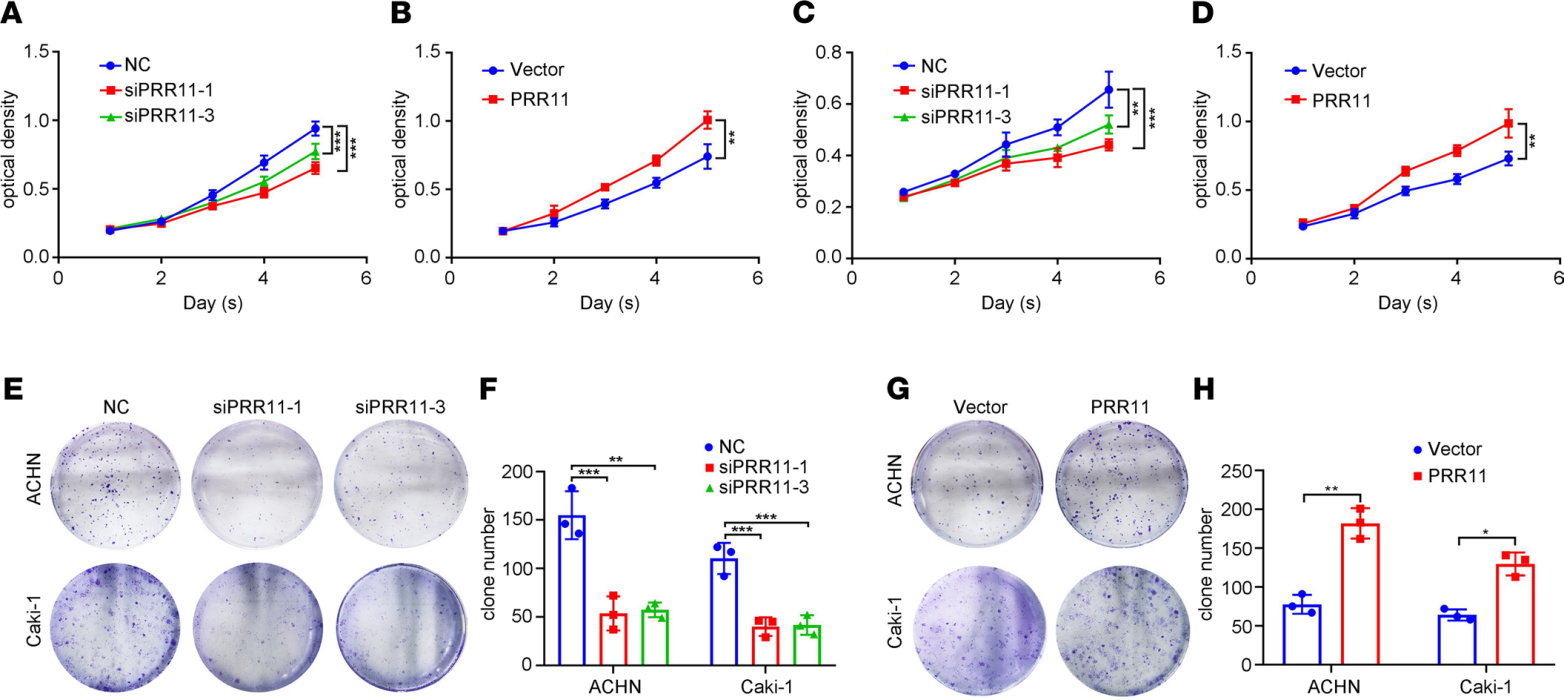
*PRR11* knockdown inhibited ccRCC cell proliferation. (**A**–**D**) MTT assay was used to evaluate the proliferation and viability of *PRR11* knockdown and PRR11-overexpressing ccRCC cells (*n* = 3). (**E**–**H**) A clonogenic assay was used to estimate the colony formation ability of *PRR11* knockdown and PRR11-overexpressing ccRCC cells, and the clone numbers were statistically analyzed (*n* = 3). The data are shown as mean ± SD. One-way ANOVA with Dunnett’s multiple comparisons test (**A**, **C**, and **F**) and 2-tailed *t* test (**B**, **D**, and **H**) analyses were performed. *P < 0.05, **P < 0.01, ***P < 0.001. PRR11, proline rich 11; ccRCC, clear cell renal cell carcinoma.

To study the relationship between PRR11 and ccRCC, we established knockdown and overexpressing ccRCC cell lines and verified the efficiency of knockdown or overexpression (Supplemental Figure 5). Next, we evaluated the proliferation of the cells. MTT and clonogenic assays suggested that *PRR11* deficiency significantly inhibited the proliferation of ACHN and Caki-1 cells, whereas PRR11 overexpression increased the proliferation of ccRCC cells (Figure 2).

**Figure 3:**
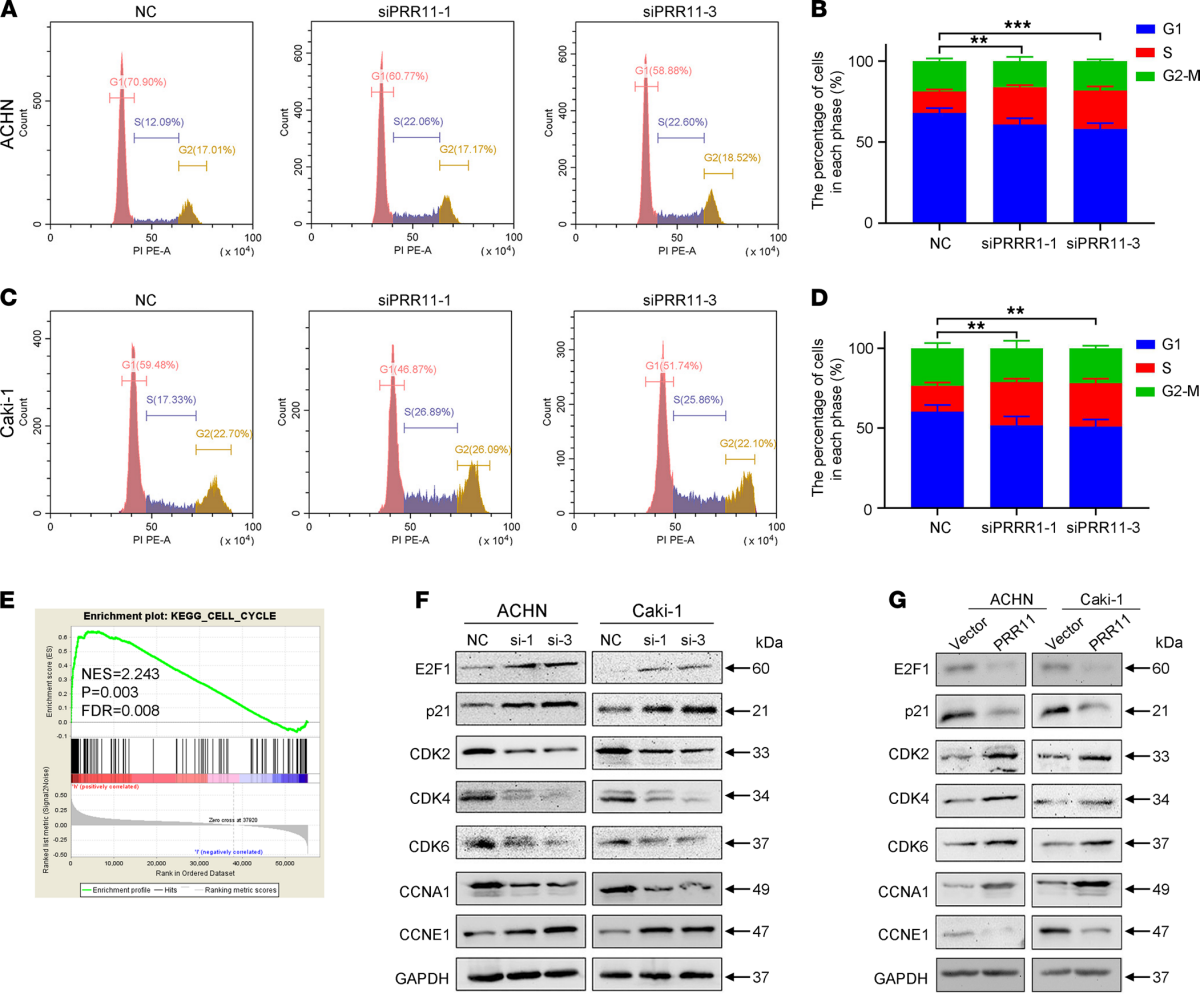
*PRR11* knockdown induced the accumulation of ccRCC cells in the S phase. (**A**–**D**) Flow cytometry analysis of the effect of *PRR11* knockdown on ccRCC cell cycle progression (*n* = 3). (**E**) GSEA was used to show the correlation between increased PRR11 expression and cell cycle progression. Nominal *P* calculated by the permutation test and FDR-corrected *q* values were obtained by the multiple hypothesis test in GSEA. (**F** and **G**) Expression of cycle-related proteins in *PRR11* knockdown or PRR11*-*overexpressing ccRCC cells via Western blotting (*n* = 3). See complete unedited blots in the supplemental material. One-way ANOVA with Dunnett’s multiple comparisons test analyses were performed. **P < 0.01, ***P < 0.001. PRR11, proline rich 11; ccRCC, clear cell renal cell carcinoma; GSEA, Gene set enrichment analysis; NES, normalized enrichment score.

Flow cytometry analysis was then performed to explore the function and role of PRR11 in ccRCC. Cell cycle analysis showed that a decrease in *PRR11* expression increased the proportion of cells in the S phase, whereas an increase in PRR11 expression resulted in a decreased proportion of cells in the S phase (Figure 3, A–D; and Supplemental Figure 6, A–D). In addition, the TUNEL assay showed that silencing *PRR11* expression increased the number of TUNEL-positive cells, and, conversely, PRR11 overexpression decreased the number of TUNEL-positive cells (Supplemental Figure 6, E–G). Consistently, flow cytometry analysis showed that silencing *PRR11* expression also induced a small amount of cell apoptosis, whereas overexpression of PRR11 decreased cell apoptosis (Supplemental Figure 7, A–F). Gene set enrichment analysis (GSEA) of 539 ccRCC samples in TCGA database showed that PRR11 expression was significantly correlated with cell cycle progression, apoptosis, DNA replication, and the p53 signaling pathway (Figure 3E and Supplemental Figure 7G).

Consistent with the flow cytometry analysis, the Western blotting results showed that silencing *PRR11* expression increased the expression of p21 and CCNE1, whereas it reduced the expression of CDK2, CDK4, CDK6, and CCNA1. In contrast, overexpression of PRR11 significantly reduced the expression of p21 and CCNE1 and increased the expression of CDK2, CDK4, CDK6, and CCNA1 (Figure 3, F and G). Simultaneously, the downregulation of *PRR11* expression could promote the phosphorylation of the DNA damage-related proteins ATM and CHK2, thereby increasing apoptosis. In contrast, when PRR11 expression was upregulated, the phosphorylation of ATM and CHK2 was reduced (Supplemental Figure 7, H and I). It is worth noting that changes in PRR11 expression significantly affected E2F1 expression in the opposite direction. Studies have shown that E2F1 can not only promote cell cycle progression to the S phase but also induce DNA damage and cause cell apoptosis (25, 31, 32), which is consistent with results of our flow cytometry analysis experiments; thus, we hypothesized that PRR11 might affect the biology of ccRCC cells by targeting E2F1 activity.

### PRR11 silencing inhibits the migration of ccRCC cells.

**Figure 4:**
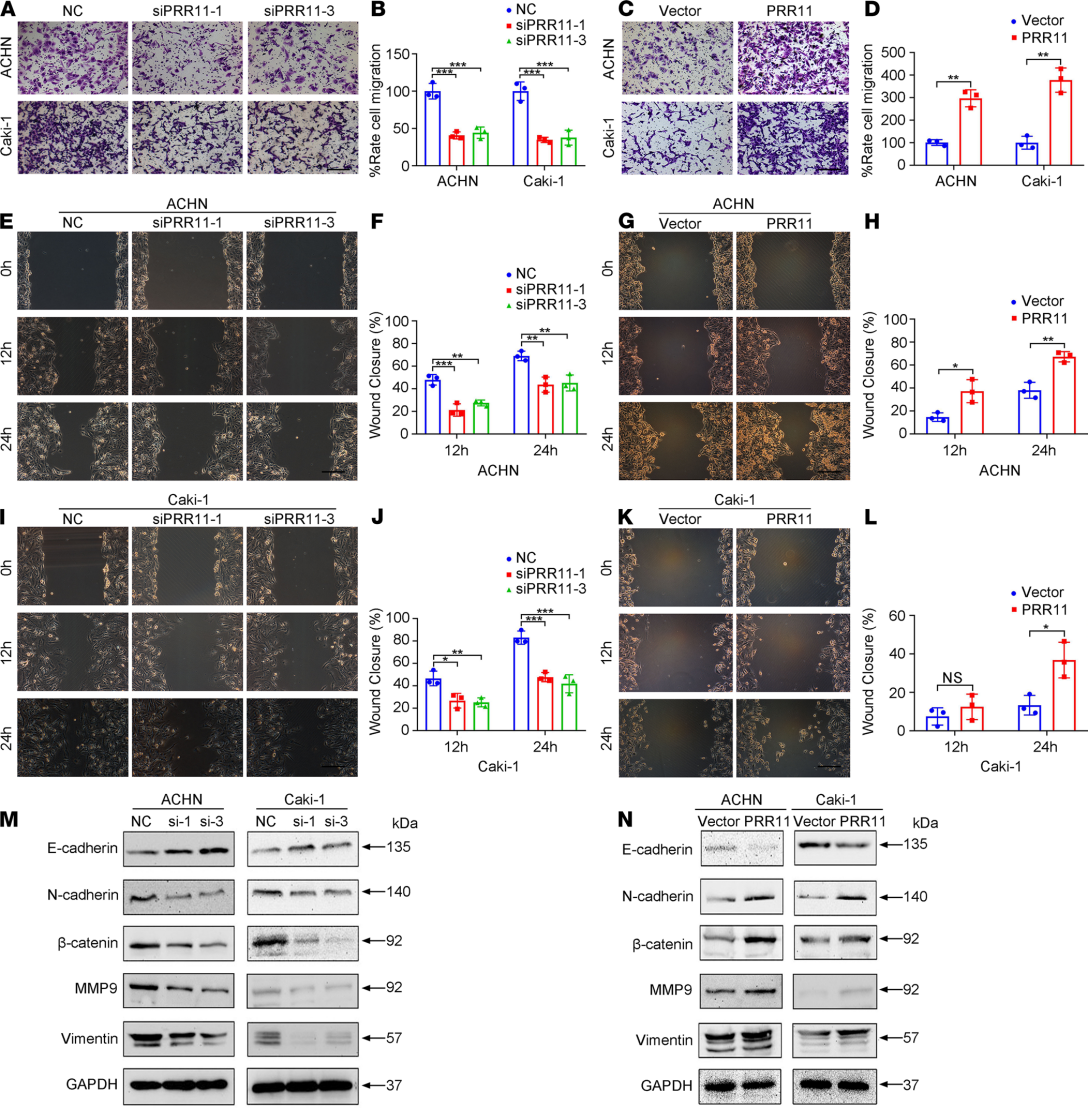
The silencing of *PRR11* expression inhibited the migration and decreased the EMT of ccRCC cells. (**A**–**D**) Transwell migration assays were used to evaluate the migration of *PRR11* knockdown and PRR11-overexpressing ccRCC cells, and the relative number of migrated cells was statistically analyzed (*n* = 3). Scale bar: 150 μm. (**E**–**L**) Wound healing assays were used to evaluate the migration of *PRR11* knockdown and PRR11-overexpressing ccRCC cells, and the relative number of migrated cells was statistically analyzed (*n* = 3). Scale bar: 150 μm. (**M** and **N**) Western blotting analysis of EMT-related protein expression in *PRR11*-silenced or *PRR11*-overexpressing ccRCC cells (*n* = 3). The data are shown as mean ± SD. One-way ANOVA with Dunnett’s multiple comparisons test (**B**, **F**, and **J**) and 2-tailed *t* test (**D**, **H**, and **L**) analyses were performed. NS, *P < 0.05, **P < 0.01, ***P < 0.001. PRR11, proline rich 11; EMT, epithelial-mesenchymal transition; ccRCC, clear cell renal cell carcinoma.

Transwell migration and wound healing analyses showed that knockdown of *PRR11* expression could reduce cell migration, whereas the upregulation of PRR11 expression promoted cell migration (Figure 4, A–L). Moreover, detection of the expression levels of E-cadherin, N-cadherin, β-catenin, Vimentin, and MMP9 by Western blotting confirmed that PRR11 promoted ccRCC cell migration (Figure 4, M and N).

In conclusion, PRR11 promoted the proliferation of ccRCC cells by influencing cell cycle progression and promoted the migration of ccRCC cells.

### Reduced PRR11 expression inhibits ccRCC cell growth and pulmonary metastasis in vivo.

**Figure 5:**
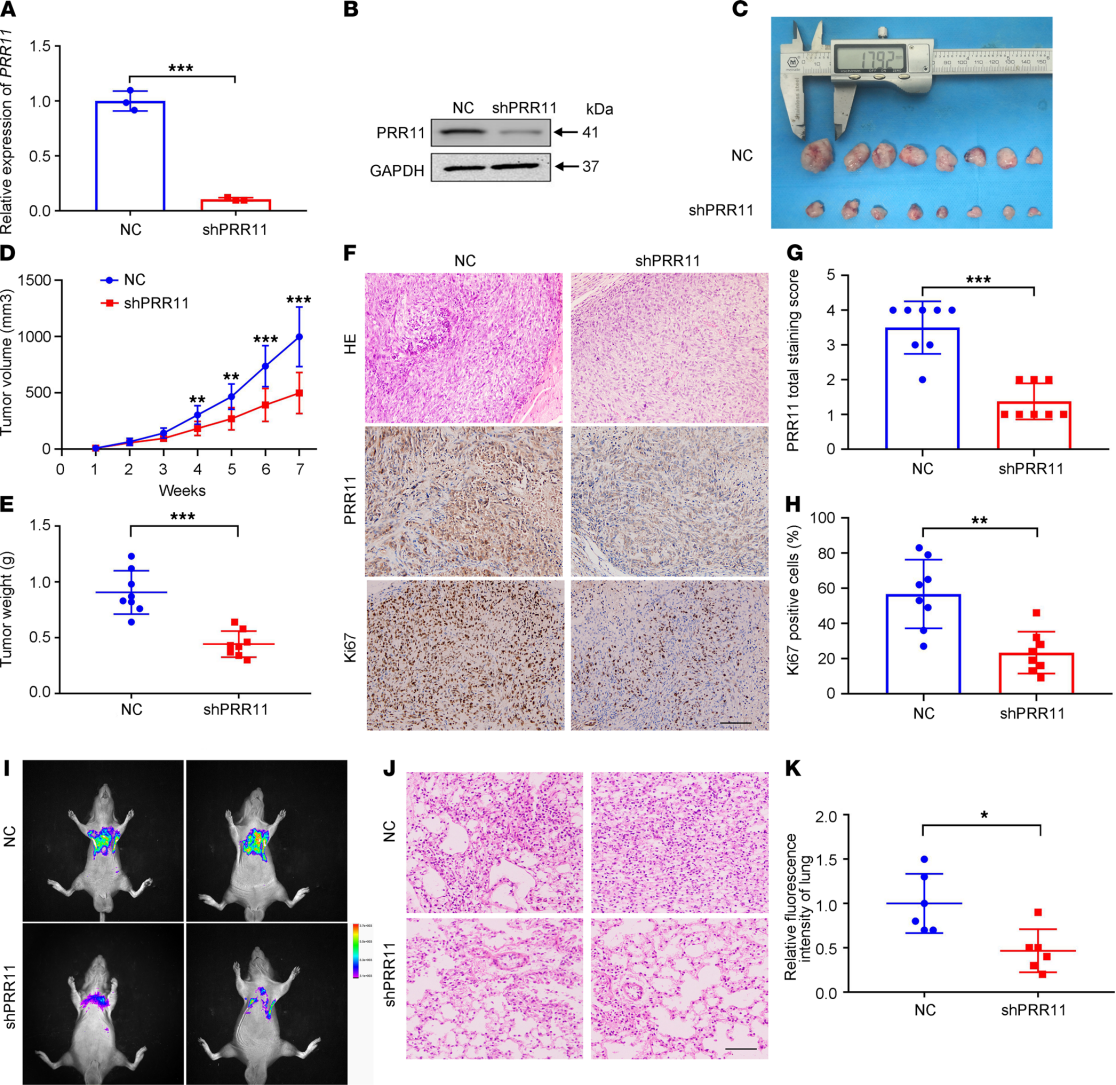
Reduction in *PRR11* expression attenuated the in vivo proliferation and migration of ccRCC cells. (**A** and **B**) The knockdown effect of LV-shPRR11 in ACHN cells was verified at the mRNA and protein levels (*n* = 3). (**C**) LV-NC and LV-shPRR11 cells were subcutaneously injected into nude mice to establish xenograft models. After 7 weeks, the xenografts were removed and photographed (*n* = 8). (**D** and **E**) The tumor volumes and weights were analyzed and evaluated (*n* = 8). (**F**) H&E staining was used to determine the degree of tumor malignancy, and IHC staining was used to evaluate the expression of PRR11 and Ki67 (*n* =8). Scale bar: 200 μm. (**G** and **H**) Quantification of the percentage of Ki67-positive cells and the PRR11 staining score (*n* = 8). (**I**–**K**) We established a lung metastasis model, detected the fluorescence intensity of the lung tumors, and performed H&E staining (*n* = 6). Scale bar: 100 μm. The data are shown as mean ± SD. Two-tailed *t* test analyses were performed. *P < 0.05, **P < 0.01, ***P < 0.001. PRR11, proline rich 11; ccRCC, clear cell renal cell carcinoma; LV-NC, lentiviral control shRNA.

After selecting stable lentiviral control shRNA (LV-NC) and LV-shPRR11 ACHN cells, the knockdown efficiency of *PRR11* was first verified (Figure 5, A and B). Next, we used stable ACHN cell lines to establish xenograft models. As shown in Figure 5, C–E, the tumor volumes and tumor weights of the LV-shPRR11 group were significantly lower than those of the LV-NC group. H&E staining showed that the LV-shPRR11 group exhibited less malignancy than the control group, and IHC staining showed that compared with the LV-NC group, the LV-shPRR11 group exhibited significantly decreased PRR11 expression and Ki67-positive cell numbers (Figure 5, F–H).

We established a lung metastasis model by caudal intravenous injection and measured the fluorescence intensity of the lung tumor cells 1 month later. As shown in Figure 5, I–K, *PRR11* knockdown significantly inhibited the migration of ccRCC cells in vivo compared with the control.

### PRR11 overexpression exerts tumorigenic effects in nonmalignant renal cells.

Next, we investigated whether PRR11 expression is associated with oncogenic phenotypes. We chose to overexpress PRR11 in the human embryonic kidney cell line 293T and the human renal tubular epithelial cell line HK2 to determine the functional role of PRR11 in nonmalignant renal cells. We selected stable LV-Vector and LV-PRR11 293T cells and HK2 cells and verified the efficiency of PRR11 overexpression (Supplemental Figure 8, A and B). MTT and clonogenic assays showed that PRR11 promoted the proliferation of nonmalignant renal cells (Supplemental Figure 8, C–F). In addition, Transwell assays showed that overexpression of PRR11 increased cell migration (Supplemental Figure 8, G and H). Flow cytometry analysis indicated that overexpression of PRR11 reduced the proportion of cells in the S phase (Supplemental Figure 8, I–L). In summary, PRR11 promoted the proliferation and migration of nonmalignant renal cells.

To explore whether PRR11 can promote tumorigenesis in vivo, we used 293T cells to establish a xenograft model in nude mice. Both the LV-Vector and LV-PRR11 groups developed tumors within the time frame of this experiment, and the tumors in the LV-PRR11 group grew more quickly (Supplemental Figure 9, A–C). In addition, histopathological analysis of the xenograft tumors showed that those in the LV-PRR11 group exhibited worse differentiation than those in the LV-Vector group (Supplemental Figure 9D).

### PRR11 is a target of c-Myc in ccRCC.

**Figure 6:**
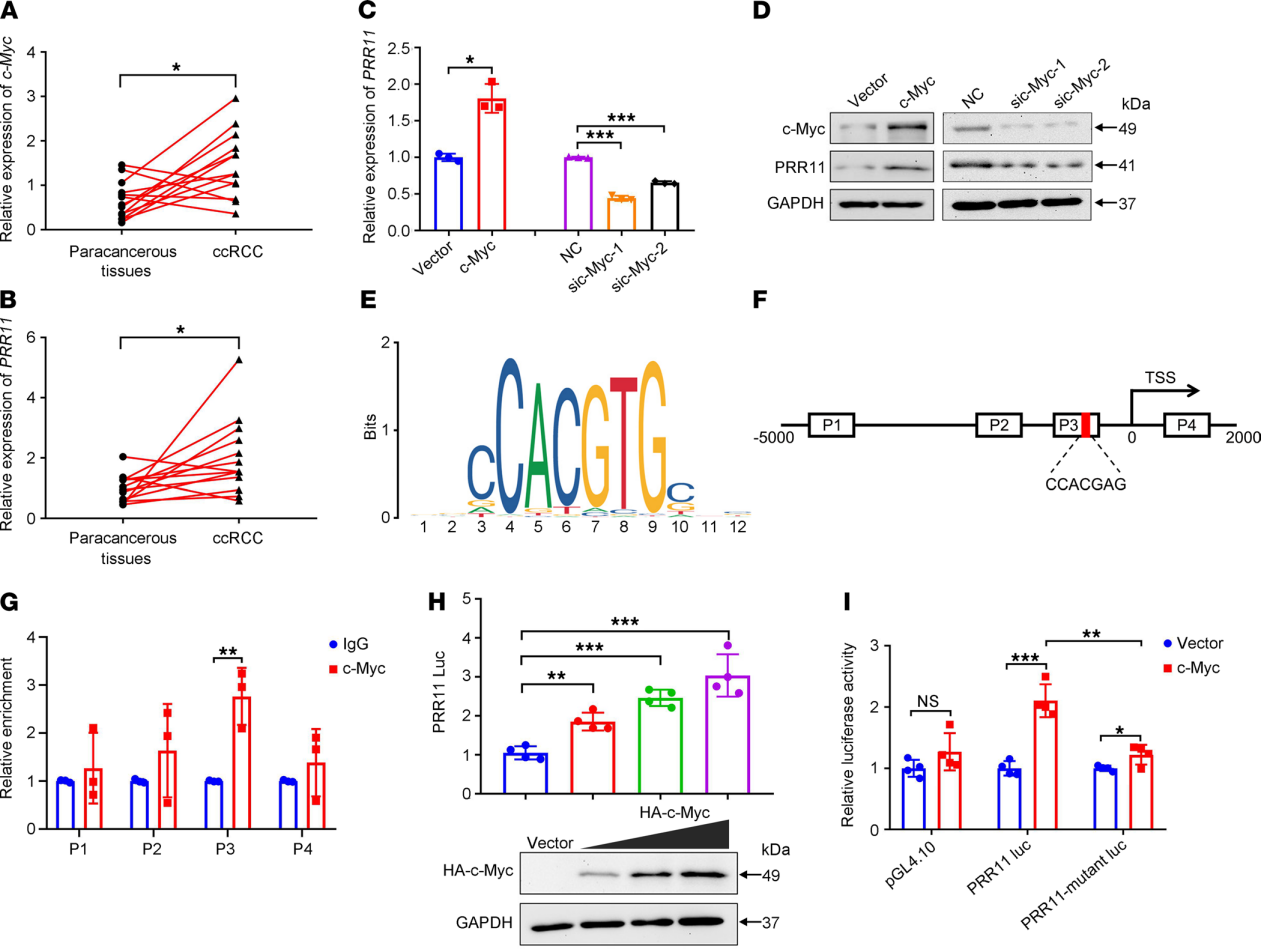
c-Myc promoted the expression of PRR11 in ccRCC cells by binding to the PRR11 promoter. (**A** and **B**) The mRNA expression levels of *PRR11* and *c-Myc* in ccRCC tissues were analyzed (*n* = 11). (**C** and **D**) The effects of changes in c-Myc expression on PRR11 expression were analyzed at the mRNA and protein levels (*n* = 3). (**E**) The binding site of c-Myc was obtained from the JASPAR database. (**F**) Schematic of the PRR11 promoter region. The box (P1-P4) indicates the sequence region covered by the ChIP-qPCR primers. The red area represents the core binding element. (**G**) ChIP-qPCR assay of the enrichment of c-Myc in the PRR11 promoter region (*n* = 3). (**H**) Luciferase activity verified that c-Myc promoted the transcription of PRR11 in 293T cells (*n* = 4). The upregulation of c-Myc expression was detected by Western blotting (*n* = 3). (**I**) A luciferase assay was performed on the WT and mutant promoters of PRR11 in ACHN cells (*n* = 4). The data are shown as mean ± SD. Two-tailed *t* test (**A**–**C**, left; and **G**), 1-way ANOVA with Dunnett’s multiple comparisons test (**C**, right; and **H**), and 2-way ANOVA with Tukey’s multiple comparisons test (**I**) analyses were performed. NS, *P < 0.05, **P < 0.01, ***P < 0.001. PRR11, proline rich 11; ccRCC, clear cell renal cell carcinoma.

c-Myc can affect the occurrence and development of different tumors through various biological pathways (33, 34). Moreover, c-Myc can also activate the transcription of many genes (35). Interestingly, c-Myc is also important for susceptibility to ccRCC (36, 37). By analyzing the public c-Myc ChIP-Seq data in the Gene Expression Omnibus (GEO) database, we found that a c-Myc peak existed in the PRR11 promoter region, proving that PRR11 may be an important target of c-Myc (Supplemental Figure 10). Therefore, we investigated and showed the regulatory effect of c-Myc on PRR11 expression in ccRCC. We first demonstrated that *c-Myc* and *PRR11* were generally highly expressed in ccRCC tissues (Figure 6, A and B). Further experiments confirmed that c-Myc could regulate the expression of PRR11 at the mRNA and protein levels in ACHN cells (Figure 6, C and D).

Next, we used the Gene-Cloud of Biotechnology Information database (https://www.gcbi.com.cn/gclib/html/index) to predict the c-Myc binding site (5′-aaCCACGTGctc-3′) in the PRR11 promoter region. JASPAR database (http://jaspardev.genereg.net/) analysis further validated the binding site of c-Myc (Figure 6E). To determine whether c-Myc binds to the PRR11 promoter region, a ChIP assay was performed in ACHN cells. As shown in Figure 6F, 4 pairs of primers were designed to cover the PRR11 promoter region and qRT-PCR was used to detect enrichment. The results showed that c-Myc was significantly enriched in the P3 region of the PRR11 promoter, but not in other regions of the PRR11 promoter (Figure 6G). Next, we performed a luciferase reporter gene analysis to confirm the targeted relationship between PRR11 and c-Myc. We transfected the empty vector and HA-c-Myc (0.5, 1, and 2 μg) into 293T cells and found that c-Myc could increase the luciferase activity of the pGL4.10-PRR11 reporter in a dose-dependent manner (Figure 6H). In addition, c-Myc significantly increased the luciferase activity of PRR11 luc in ACHN cells, whereas mutant PRR11 luc constructs significantly reduced this increased luciferase activity (Figure 6I).

### PRR11 attenuates E2F1 protein stability by interacting with E2F1.

**Figure 7:**
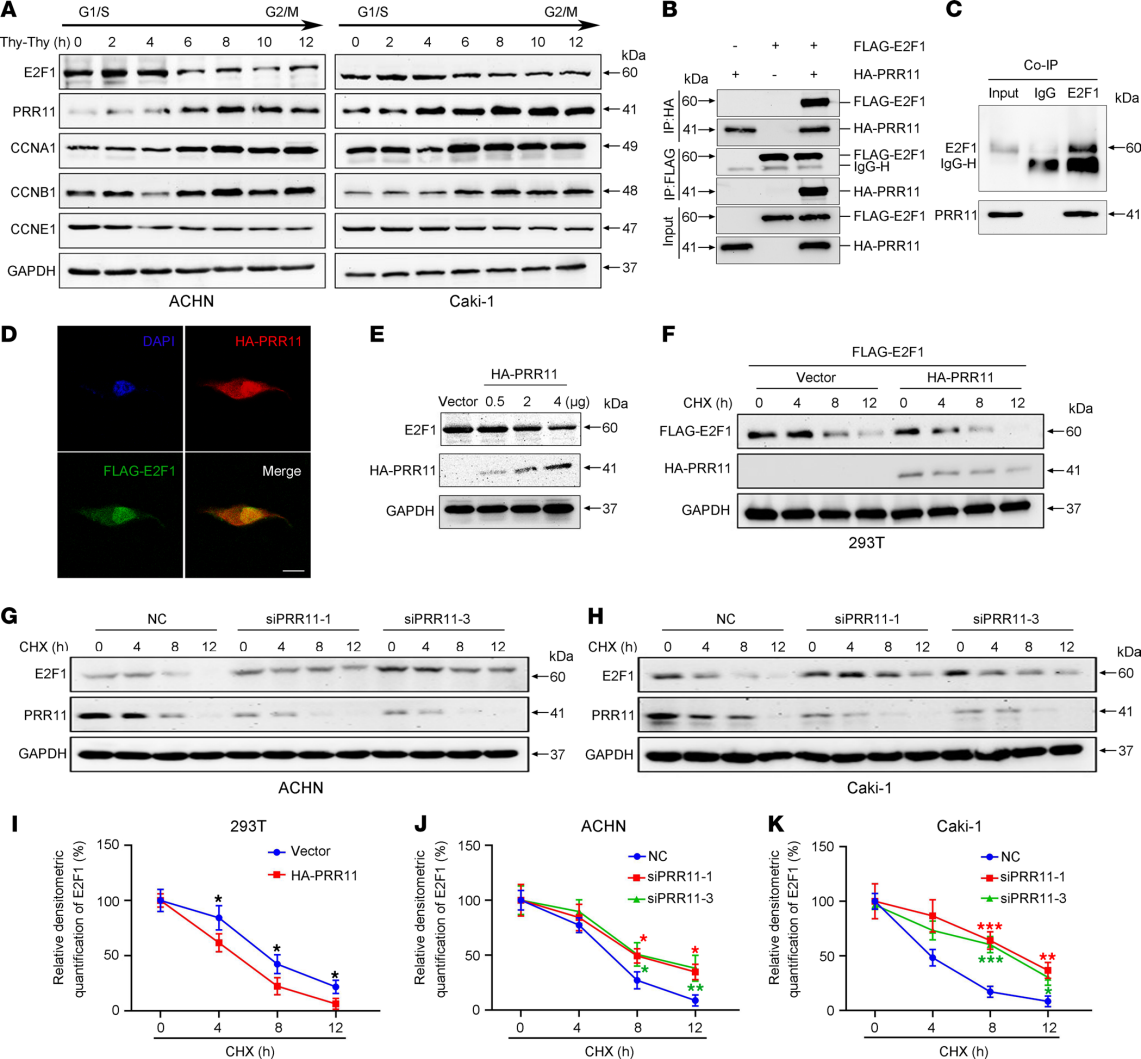
PRR11 reduced the stability of the E2F1 protein by interacting with E2F1. (**A**) Western blotting analysis of ACHN cells and Caki-1 cells subjected to double thymidine blocking (*n* = 3). (**B**) Co-IP assay of exogenous E2F1 protein and PRR11 protein was performed in 293T cells (*n* = 3). (**C**) Co-IP assay of endogenous E2F1 protein and PRR11 protein was performed in ACHN cells (*n* = 3). (**D**) The colocalization of PRR11 and E2F1 in ACHN cells was analyzed by observing fluorescence signals by confocal immunofluorescence microscopy. Scale bar: 15 μm. (**E**) The effect of PRR11 on E2F1 protein levels in ACHN cells was analyzed by Western blotting (*n* = 3). (**F**–**H**) The effect of PRR11 on E2F1 protein degradation was investigated with the exogenous and endogenous expression of the proteins by an CHX assay (*n* = 3). (**I**–**K**) Quantification of E2F1 protein degradation rate (*n* = 3). Two-tailed *t* test (**I**) and 1-way ANOVA with Dunnett’s multiple comparisons test (**J** and **K**) analyses were performed. *P < 0.05, **P < 0.01, ***P < 0.001. PRR11, proline rich 11; Co-IP, coimmunoprecipitation; CHX, cycloheximide.

To study the relationship between PRR11 and E2F1 in ccRCC, we first performed a cell cycle synchronization experiment to assess whether these molecules are related to the cell cycle. The cycle synchronization results showed that E2F1 was highly expressed in cells in the G1/S phase, whereas E2F1 was expressed at relatively low levels in cells in the G2/M phase. In contrast, PRR11 was highly expressed in cells in the G2/M phase, whereas its expression was reduced in cells in the G1/S phase (Figure 7A). These results suggested that there may be some association between PRR11 and E2F1 by which these molecules jointly regulate cell cycle progression.

Next, we confirmed the protein-protein interaction between both endogenous and exogenous PRR11 and E2F1 with coimmunoprecipitation (Co-IP) experiments (Figure 7, B and C). Immunofluorescence analysis of colocalization showed that both PRR11 and E2F1 were expressed in the nucleus and cytoplasm, but they were mainly expressed in the nucleus (Figure 7D). To further explore the profound relationship between these molecules, we performed experiments at the protein and mRNA levels. The results showed that the downregulation of *PRR11* expression did not cause any change in *E2F1* mRNA expression (Supplemental Figure 11A), whereas the increase in PRR11 expression significantly reduced the protein expression of E2F1 in a dose-dependent manner (Figure 7E). Therefore, we hypothesized that PRR11 might affect cell cycle progression by regulating the stability of the E2F1 protein. To investigate the effect of PRR11 on the stability of the E2F1 protein, we performed a cycloheximide (CHX) assay with both exogenous and endogenous protein expression. When HA-PRR11 and Flag-E2F1 were overexpressed in 293T cells, we found that exogenous PRR11 could accelerate the degradation rate of exogenous E2F1. Consistently, the lack of endogenous PRR11 expression led to slower degradation of endogenous E2F1 (Figure 7, F–K). Together, these results indicate that PRR11 interacted with the E2F1 to attenuate the stability of E2F1 protein in order to affect the progression of the cell cycle and thus promote the development of ccRCC.

### Downregulation of E2F1 expression partially rescues the inhibition of ccRCC caused by PRR11 deficiency.

**Figure 8:**
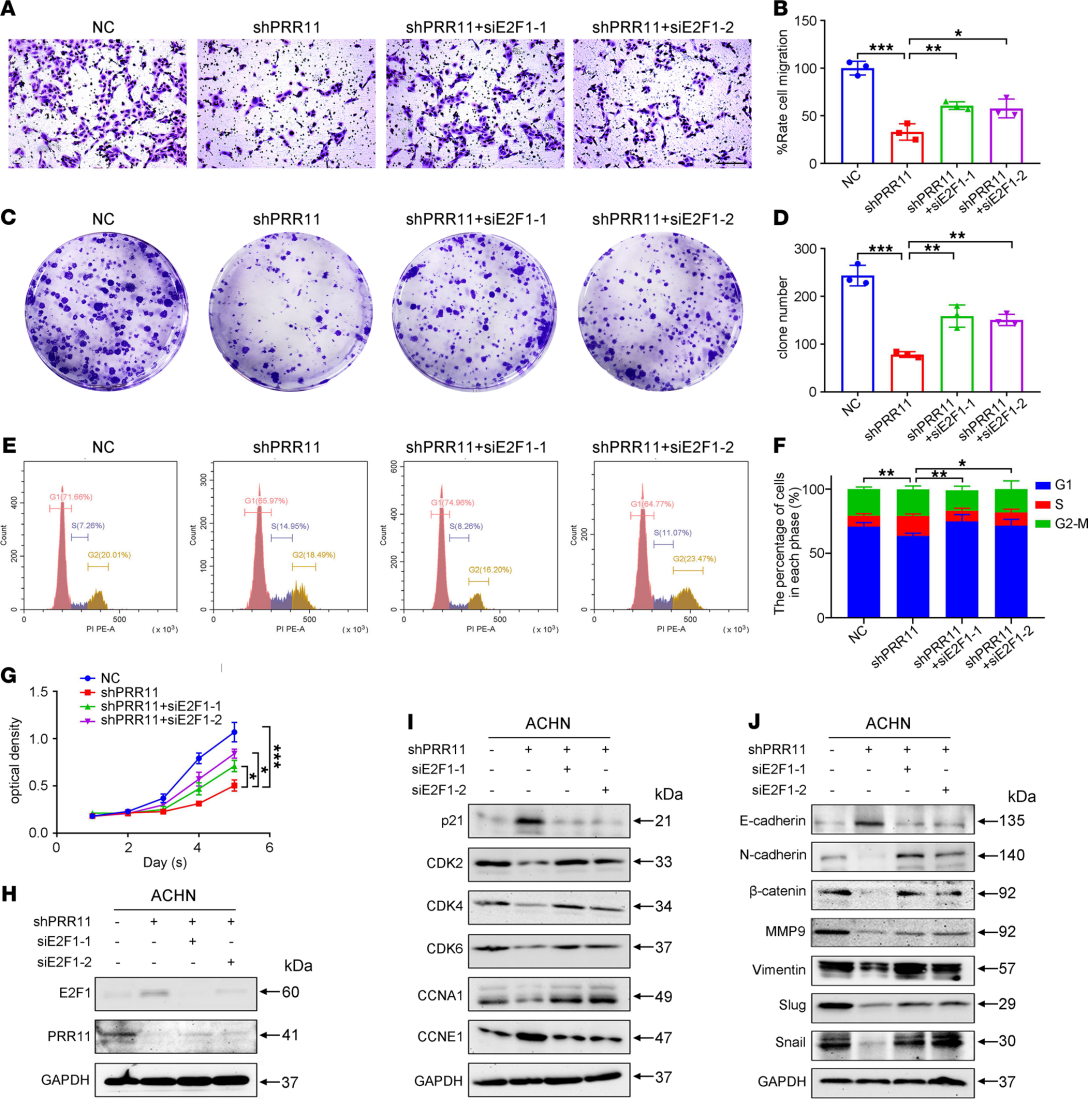
E2F1 played an important role in the PRR11-mediated regulation of the proliferation and migration of ccRCC cells. (**A**–**D**) Transwell and clonogenic assays were used to analyze the effect of *E2F1* knockdown on the migration and colony formation of ACHN cells transfected with shPRR11 (*n* = 3). Scale bar: 150 μm. (**E** and **F**) *E2F1* knockdown significantly reversed the effect on the cell cycle progression of shPRR11-transfected ACHN cells (*n* = 3). (**G**) MTT assay confirmed that *E2F1* knockdown significantly enhanced the proliferation of ACHN cells transfected with shPRR11 (*n* = 3). (**H**–**J**) The expression of PRR11, E2F1, cell cycle–related genes, and migration-related genes in ccRCC cells was determined by Western blotting. The data are shown as mean ± SD. Two-way ANOVA with Tukey’s multiple comparisons test analyses were performed (*n* = 3). *P < 0.05, **P < 0.01, ***P < 0.001. ccRCC, clear cell renal cell carcinoma; PRR11, proline rich 11.

To study the combined effect of PRR11 and E2F1 in ccRCC, we established 4 groups for subsequent cellular function experiments. Transwell migration assays indicated that *E2F1* downregulation rescued the shPRR11-mediated inhibition of ccRCC cell migration (Figure 8, A and B). Clonogenic assays showed that *E2F1* downregulation reversed the inhibition of colony formation that caused *PRR11* deletion (Figure 8, C and D). In addition, flow cytometry analysis indicated that compared with the NC-transfected group, the shPRR11-transfected group exhibited a significantly increased proportion of cells in the S phase, whereas the proportion of cells in the S phase was significantly reduced in the shPRR11 and siE2F1-1 and shPRR11 and siE2F1-2 transfection groups (Figure 8, E and F). qRT-PCR analysis of corresponding cell cycle–related genes was also conducted (Supplemental Figure 11, B and C). MTT assays demonstrated that *E2F1* downregulation reversed the shPRR11-mediated inhibition of ccRCC cell proliferation (Figure 8G). In addition, downregulation of *E2F1* expression reversed the shPRR11-mediated promotion of ccRCC cell apoptosis (Supplemental Figure 11, D and E). Then, the expression of cell cycle–, apoptosis-, and migration-related proteins in each group was analyzed, and the results confirmed our hypothesis (Figure 8, H–J, and Supplemental Figure 11F). The experimental results described above demonstrated that PRR11 accelerated the progression of ccRCC by inactivating E2F1.

## Discussion

The cell cycle is an ordered set of events that eventually lead to cell growth and division. The stable progression of the cell cycle is important for the maintenance of normal human function, and disordered cell cycle progression has been proved to be the initiating factor for ccRCC occurrence (38). Growing evidence indicates that the activation of oncogenes or the inactivation of tumor suppressor genes can affect cell cycle progression and cause tumor occurrence and development (39, 40).

Our study first identified PRR11 as a potentially novel biomarker of ccRCC through public databases and tissue microarrays, and this study showed that PRR11 is an independent and unfavorable prognostic factor of ccRCC. PRR11 has been widely reported in many tumor types, but its biological function has not been studied in depth. The biological role of PRR11 in ccRCC is of great interest to us.

Here, we demonstrated that PRR11 functions as a tumor-promoting factor in ccRCC. In addition, the potential of PRR11 to initiate tumor cell proliferation and migration in ccRCC depends on the novel function of the classic transcription factor E2F1. Considering that PRR11 and E2F1 exert opposite effects on the progression of cells through the S phase and the diametrically opposite changes in the protein levels in the cell cycle synchronization experiments, we hypothesized that PRR11 might interact with E2F1, thereby affecting ccRCC cell cycle progression. To further validate this hypothesis, Co-IP and colocalization experiments were performed to confirm that PRR11 could bind to E2F1 and thus affect E2F1 activation. In addition, we added CHX to the medium of 293T, ACHN, and Caki-1 cell cultures and validated the effect of PRR11 on the stability of the E2F1 protein through experiments focusing on both endogenous and exogenous protein expression. Many studies have confirmed the cancer-promoting effect of E2F1. E2F1 can promote the proliferation of tumor cells and reduce the sensitivity of tumor cells to radiotherapy and chemotherapy (41–44). Paradoxically, E2F1 can also inhibit tumor growth by inducing cell senescence and apoptosis (45–49). Hence, E2F1-mediated tumor-related events may be unstable and variable, and they may alter tumor development in response to certain factors. It has been demonstrated in RCC that the loss of *VHL* gene function leads to the accumulation of E2F1, which promotes cell senescence and thus inhibits tumor progression (24). Therefore, we hypothesized that the inhibition of ccRCC caused by the downregulation of *PRR11* expression might occur due to the excessive accumulation of E2F1, which leads to cell senescence.

Multiple studies have shown that the transcription factor c-Myc acts as a protooncogene in ccRCC (50). c-Myc promotes the development of ccRCC by regulating glucose metabolism (51). We first demonstrated that both *c-Myc* and *PRR11* expression were markedly upregulated in ccRCC tissues. It was then confirmed that c-Myc promoted the transcription of PRR11. Finally, we performed ChIP assay and gene promoter luciferase reporter analysis to prove that c-Myc was a transcription factor that regulated PRR11 expression.

PRR11 accelerated the development of ccRCC by promoting cell proliferation and migration. Furthermore, PRR11 reduced E2F1 expression by interacting with the E2F1 protein and reducing its stability in ccRCC cell lines. In addition, we discovered a new transcription factor that regulated PRR11 expression, namely, c-Myc, and overexpression of c-Myc promoted PRR11 expression in ccRCC cells by binding to the PRR11 promoter region (5*′*-aaCCACGTGctc-3*′*). However, the detailed biophysical mechanisms remain to be explored. Most studies have shown that E2F1 activity is regulated by proteolysis that is mediated by the ubiquitin-proteasome system (52–56). Therefore, we hypothesize that PRR11 may affect the degradation of E2F1 by regulating a certain association between E2F1 ubiquitination and deubiquitination, thereby affecting the senescence of ccRCC cells. In short, our research team will continue to study the correlation between PRR11 and E2F1 expression in ccRCC and potential problems that occur as a result.

In summary, we report the function of a gene in the occurrence of ccRCC. PRR11 may have been a potential target for the treatment of ccRCC in the clinic treatment and may have promoted tumor development by affecting the stability of E2F1.

## Methods

### Bioinformatics analysis.

We collected ccRCC microarray data from the TCGA database (https://cancergenome.nih.gov/) and then used the “limma” R package to normalize the microarray data to eliminate data bias. Based on the collected data, we conducted Cox analyses and constructed a nomogram. Then, calibration curves and ROC curves were used to test the performance of the nomogram. We used the clinical data of the microarray to determine the PRR11 mRNA expression in samples with different histological grades or pathological stages. Taking the median expression level of PRR11 as the cutoff point, 539 samples in the ccRCC microarray were divided into PRR11 high expression group and PRR11 low expression group, and then survival analysis and GSEA were performed. We chose annotated gene sets c2.cp.kegg. V7.4.symbols.gmt as the reference. Gene sets with an FDR < 0.05 were considered to be significantly enriched (ref. 57; GSEA, http://software.broadinstitute.org/gsea/index.jsp).

### Plasmid construction.

We first synthesized PRR11 cDNA by PCR technology and cloned it into the pcDNA3-2×Flag and pcDNA3-HA empty vectors. The PRR11 forward primer was 5*′*-CATGCCATGGCCAAGTTCAAACAACGAAGACGA-3*′*, and the reverse primer was 5*′*-ATAAGAATGCGGCCGCGTTTTGTTCATCAAAGCTGCTTG-3*′*. Sequencing was performed to verify the DNA sequence. We obtained the pGL4.10-PRR11 promoter plasmid from Obio Technology Corp., and we synthesized the mutant pGL4.10-PRR11 promoter plasmid.

### Cell culture and transfections.

The HK2 cell line was provided by Stem Cell Bank, Chinese Academy of Sciences (Shanghai, China). The 293T, 786-O, 769-P, ACHN, and Caki-1 cell lines were provided by Cell Bank, Chinese Academy of Sciences (Shanghai, China). The HK2 and 293T cell lines were maintained in DMEM; the 786-O, and 769-P cell lines were maintained in RPMI 1640; the ACHN cell line was maintained in MEM; and the Caki-1 cell line was maintained in McCoy’s 5A. All the cell lines were cultured in medium supplemented with 10% FBS.

We purchased siPRR11, siE2F1, sic-Myc, and siVHL from GenePharma. The siRNA sequences targeting PRR11, E2F1, c-Myc, and VHL were as follows: siPRR11-1 (si-1)/shPRR11*:* 5′-ACGCAGGCCUUAAGGAGAATT-3′; siPRR11-2 (si-2): 5′-GCACGGAAUCCACUAGUUATT-3′; siPRR11-3 (si-3): 5′-GGCCUUAAGGAGAAAGUUUTT-3′; siE2F1-1: 5′-GCGCAUCUAUGACAUCACCTT-3′; siE2F1-2: 5′-GGACUCUUCGGAGAACUUUTT-3′; siVHL-1: 5′-CGAGCGCGCGCGAAGACUACG-3′; siVHL-2: 5′- CCAAUGGAUUCAUGGAGUA-3′; siVHL-3: 5′- GGAGCGCAUUGCACAUCAA-3′; sic-Myc-1: 5′- GCUUGUACCUGCAGGAUCUTT-3′; and sic-Myc-2: 5′-GGAAGAAAUCGAUGUUGUUTT-3′. Lipofectamine 3000 (Invitrogen) was used for cell transfection.

We purchased LV-NC, LV-shPRR11, LV-Vector, and LV-PRR11 lentivirus from GenePharma and selected the lentivirus-infected cells with puromycin (MilliporeSigma) to obtain stable puromycin-resistant cell lines.

### Cell phenotype experiment.

For the MTT assay, the cells (3000/well) were plated in 96-well plates, and the absorbance at different time points was measured to evaluate cell viability.

To perform the clonogenic assay, the cells (2000/well) were plated in a 6-well plate, fixed after 14 days, and stained with crystal violet.

For the Transwell migration assay, 30,000 cells were plated in serum-free medium in the upper chambers (Corning), and then migration into the lower chambers was induced with serum. The cells were fixed and stained after 24 hours. Three random fields in each well were photographed, and the migrated cells were counted with NIH ImageJ software.

For the wound healing assay, we wounded the cell cultures with a sterile 200 μL pipette tip and washed the cells with PBS. Images of the same wounded areas were captured at 0, 12, and 24 hours after wounding. The percentage of wound closure was quantified with NIH ImageJ software.

### Flow cytometry analysis.

The ccRCC cells were incubated with 1× DNA Staining Solution, which contained propidium iodide and permeabilization solution (Multisciences), for half an hour before cell cycle analysis. For apoptosis analysis, the cells were prepared using the FITC Annexin V Apoptosis Detection Kit I (BD Biosciences) and then analyzed by flow cytometry.

### qRT-PCR.

Total RNA was extracted using HiPure Total RNA Mini Kit (Magen, R4111-03), and the RNA was reverse-transcribed into cDNA. qRT-PCR was performed using iQ SYBR Green Supermix (Bio-Rad). The primer sequences are listed in Supplemental Table 2.

### Western blotting and immunofluorescence staining.

The cells were lysed in RIPA buffer (MilliporeSigma) with phosphatase inhibitors and protease inhibitors, and the supernatants were collected by high-speed centrifugation. Protein extracts were loaded into SDS-PAGE gels for separation, transferred to PVDF membranes, and incubated with antibodies twice after being blocked with 5% milk. Electromagnetic interference XRS Imaging System (Bio-Rad) was used to detect proteins. The primary and secondary antibody information is provided in Supplemental Table 3. Immunofluorescence staining was performed by Biofavor Biotechnology, and the samples were analyzed by confocal microscopy.

### TUNEL assay.

The fixed cells were permeabilized with 0.1% Triton X-100. Then, cells were labeled using the TUNEL kit (Roche Applied Science). The numbers of apoptotic cells were detected and quantified by using a confocal microscope and NIH ImageJ software.

### Co-IP assay and CHX assay.

First, 20 μL protein A/G magnetic beads were fully resuspended and coincubated with 1 μg target antibody for 4 hours. Then, it was added into the cell lysates and incubated overnight at 4°C. The magnetic bead–coupled complex was washed several times with IP buffer. Finally, 1× SDS buffer solution was used to elute the protein-antibody-bead complexes and Western blotting analysis was conducted. CHX (200 μg/mL, MCE, HY-12320) was added to the ccRCC cells to detect the stability of the proteins at different time points.

### ChIP assay.

IGV software was used to analyze the c-Myc ChIP-Seq data in the GEO database with accession number GSE138295, and putative binding sites of c-Myc and PRR11 were identified. A ChIP assay was performed with a Simple ChIP Plus sonication Chromatin IP Kit (Cell Signaling Technology, 56383) and according to the manufacturer’s protocol. Briefly, formaldehyde–cross-linked ACHN cells were lysed with nuclear lysis buffer and then sonicated with Bioruptor (8 cycles; sonication for 30 seconds, followed by rest for 90 seconds). The sizes of the DNA fragments were analyzed by agarose gel electrophoresis, and the sizes were determined to be approximately 100–1000 bp. Chromatin immunoprecipitation was performed with anti–c-Myc or rabbit IgG antibodies. The eluted and purified DNA was analyzed by qRT-PCR. Information about the primers specific for the PRR11 promoter is provided in Supplemental Table 4.

### Luciferase reporter assay and cell synchronization.

Target cells were cotransfected with empty vector, pGL4.10-PRR11 promoter plasmid, mutated pGL4.10-PRR11 promoter plasmid, and c-Myc overexpression plasmid. Reporter gene activity was assessed using the Dual-Luciferase Reporter Assay System (Promega, E1910). To detect the changes in PRR11 and E2F1 expression in cells undergoing cell cycle progression changes, we used a double thymidine (2 mM; MilliporeSigma, T1895) block to arrest the cells in the G1/S phase. Protein levels of E2F1 and PRR11 at different time points were detected by Western blotting.

### IHC staining.

A ccRCC tissue microarray including 90 ccRCC tissues and 90 paracancerous tissues was purchased from Shanghai Outdo Biotech. In addition, 13 pairs of ccRCC and paracancerous tissues were collected from our hospital. In general, we hydrated paraffin sections, embedded them, incubated them with 3% H_2_O_2_, and then incubated them with citrate buffer to retrieve the antigens. The tissues were blocked with 5% BSA and incubated with primary and secondary antibodies. Finally, the tissues were successively incubated with HRP substrate solution and DAB substrate chromogen solution. We scored the intensity of PRR11 staining as 0, 1, 2, or 3 and divided the samples into high (3 and 4) and low (1 and 2) groups according to the staining intensity score. The expression of PRR11 in the tissue microarrays was blindly assessed by 2 pathologists. To quantify Ki67 levels by IHC staining, NIH ImageJ software was used for the quantification, and the percentage of Ki67-positive cells was calculated based on the total number of nuclei.

### Xenograft and lung metastasis models.

We purchased male BALB/c nude mice (6 weeks old) from Beijing HFK Bioscience Co. and fed them adaptively for 1 week in the animal laboratory of Zhongnan Hospital. LV-NC or LV-shPRR11 ACHN cells (1 × 10^6^ cells/150 μL) were subcutaneously implanted into the nude mice (*n* = 8). Due to the low tumorigenicity of 293T cells, Matrigel (Corning) was chosen as a cell carrier for cell transplantation to improve the survival rate of the cells after implantation. LV-Vector or LV-PRR11 293T cells (5 × 10^6^ cells/150 μL) were suspended in a PBS and Matrigel mixture (1:1) and implanted into the nude mice (*n* = 8). The tumor volume (V) was measured with a Vernier caliper and calculated according to the standard formula V = 0.5 × length × width^2^. To verify the effect of PRR11 on migration in vivo, LV-NC or LV-shPRR11 ACHN cells (1 × 10^6^ cells/100 μL) were injected into the tail vein of the mice (*n* = 6), and the fluorescence intensity of the lung metastases in the nude mice was measured. The tumor and lung tissues were carefully harvested, weighed, and fixed with 4% PFA for subsequent analysis.

### Statistics.

All the results were obtained from more than 3 independent experiments. GraphPad Prism 7 was used to analyze the data, and statistical differences were analyzed by 2-tailed *t* test, 1-way ANOVA test, and 2-way ANOVA test. All results are presented at the statistical threshold of a *P* value less than 0.05.

### Study approval.

The Ethics Committee of Zhongnan Hospital of Wuhan University approved the use of the ccRCC tissues and adjacent tissues (approval number 2020102), and informed consent was obtained from all the subjects. The animal studies were approved by the Institutional Animal Care and Use Committee at Center for Animal Experiment, Wuhan University, and were carried out in accordance with the *Guide for the Care and Use of Laboratory Animals* (National Academies Press, 2011).

## Author contributions

SC designed and conducted the experiments and drafted the manuscript. ZH conducted experiments and the statistical analyses and edited the manuscript. SC and ZH contributed equally to this work. TP and FZ conducted the experiments. LJ, YX, GW, and KQ reviewed and edited the manuscript. LJ and YX conducted all the statistical analyses. XW supervised and designed the experiment. All the authors read and approved the final manuscript.

## Supplementary Material

Supplemental data

## Version 1 09/09/2021

In-Press Preview

## Version 2 10/08/2021

Electronic publication
