# Supplementary material for: PRR11 promotes ccRCC tumorigenesis by regulating E2F1 stability
Source: JCI Insight. 2021 Oct 8;6(19):e145172. doi: 10.1172/jci.insight.145172 (PMC8525590; doi:10.1172/jci.insight.145172)

Supplementary Information

Supplementary Figures

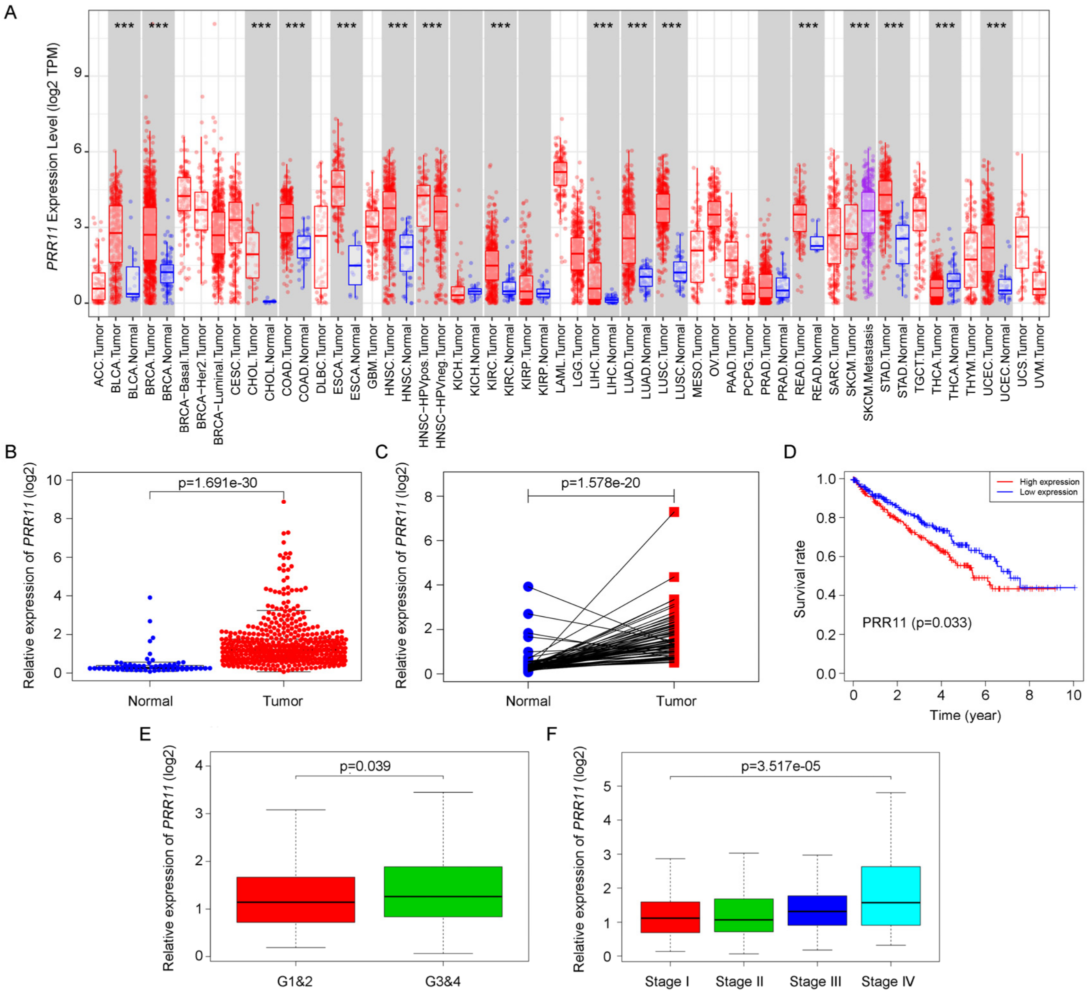

Supplementary Figure S1. Bioinformatics analyses of *PRR11* expression in TCGA. (A) *PRR11* expression in different tumor types in the TIMER database. **(B)** In TCGA database, *PRR11* mRNA was overexpressed in unpaired ccRCC samples (n = 72 for normal samples and n = 539 for tumor samples). **(C)** In TCGA database, *PRR11* mRNA was overexpressed in ccRCC paired samples (n = 72). **(D)** Kaplan-Meier survival analysis of ccRCC patients based on TCGA database. **(E-F)** *PRR11* expression was positively correlated with tumor grade (n = 243 for G1&2 and n = 279 for G3&4) and tumor stage (n = 265 for Stage I, n = 57 for Stage II, n = 123 for Stage III and n = 82 for Stage IV) in ccRCC tissues. The microarray data of TCGA dataset were normalized using the "limma" R package. The data are shown as the mean ± SD. Two-tailed *t*-test **(A-C, E)** and one-way ANOVA **(F)** test analyses were performed.

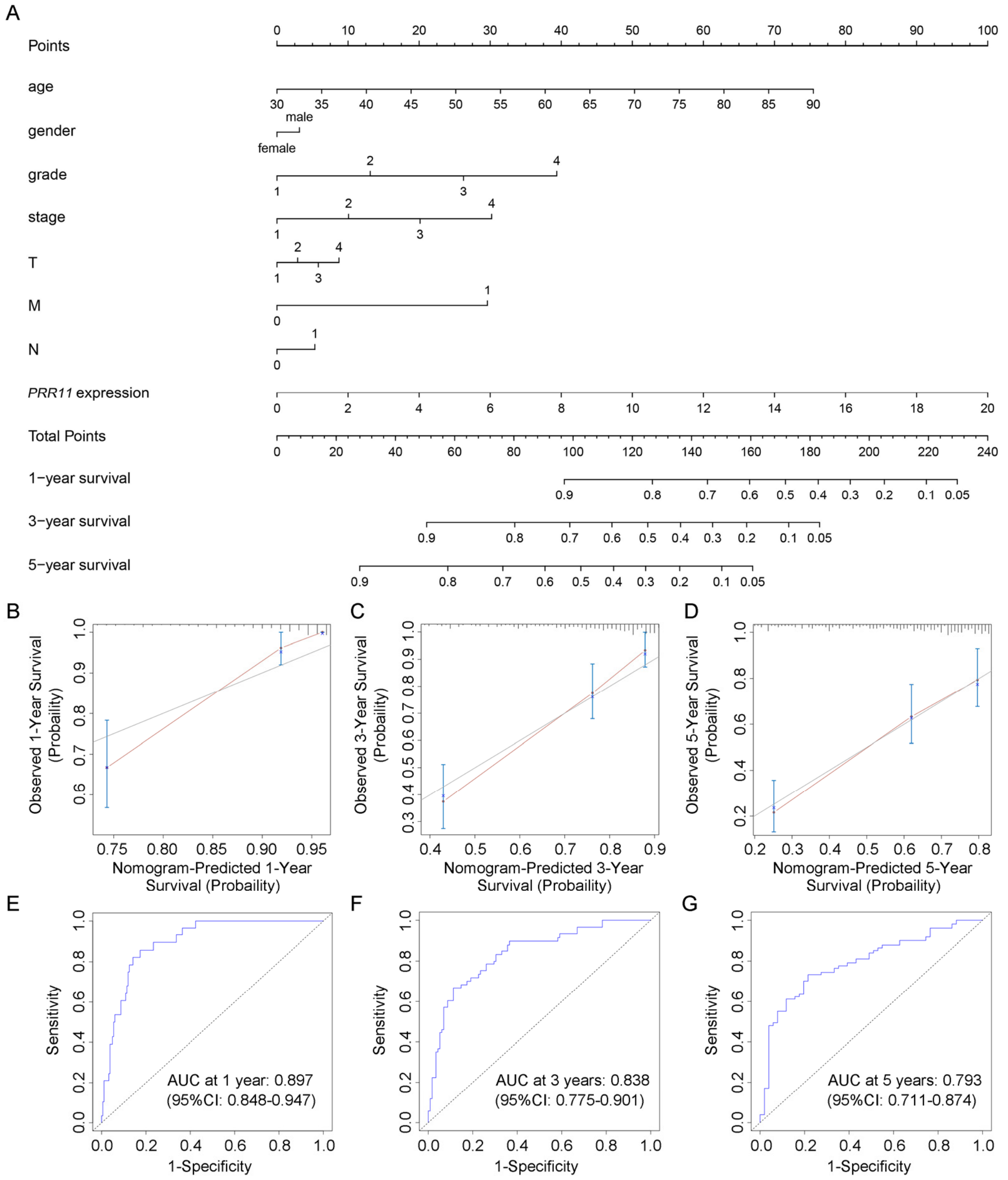

Supplementary Figure S2. Predictive nomogram and calibration curves based on TCGA database. (A) The nomogram for predicting 1-, 3-, and 5-year survival in ccRCC patients. **(B-D)** Generation of the nomogram's 1-, 3-, and 5-year calibration curves. **(E-G)** Generation of the nomogram's 1-, 3-, and 5-year ROC curves.

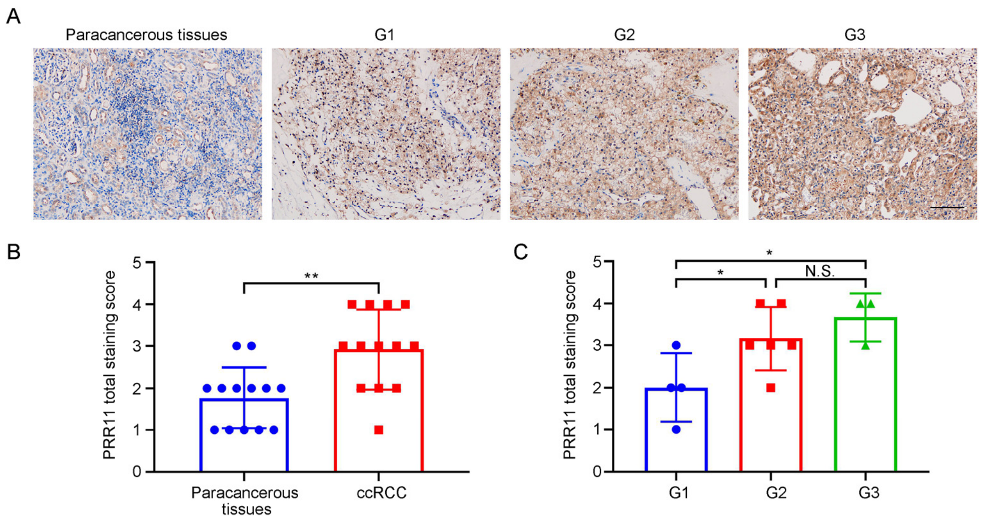

Supplementary Figure S3. IHC staining of samples from our hospital to detect PRR11 expression. (A-C) IHC staining indicated that the expression of PRR11 in ccRCC tissues was significantly higher than that in normal kidney tissues (n = 13), while the expression of PRR11 was higher in tumors of lower grades (n = 4 for G1, n = 6 for G2 and n = 3 for G3). Scale bar: 200 μm. The data are shown as the mean ± SD. Two-tailed *t*-test **(B)** and one-way ANOVA test **(C)** analyses were performed. N.S. = No significance, * $p < 0.05$, ** $p < 0.01$.

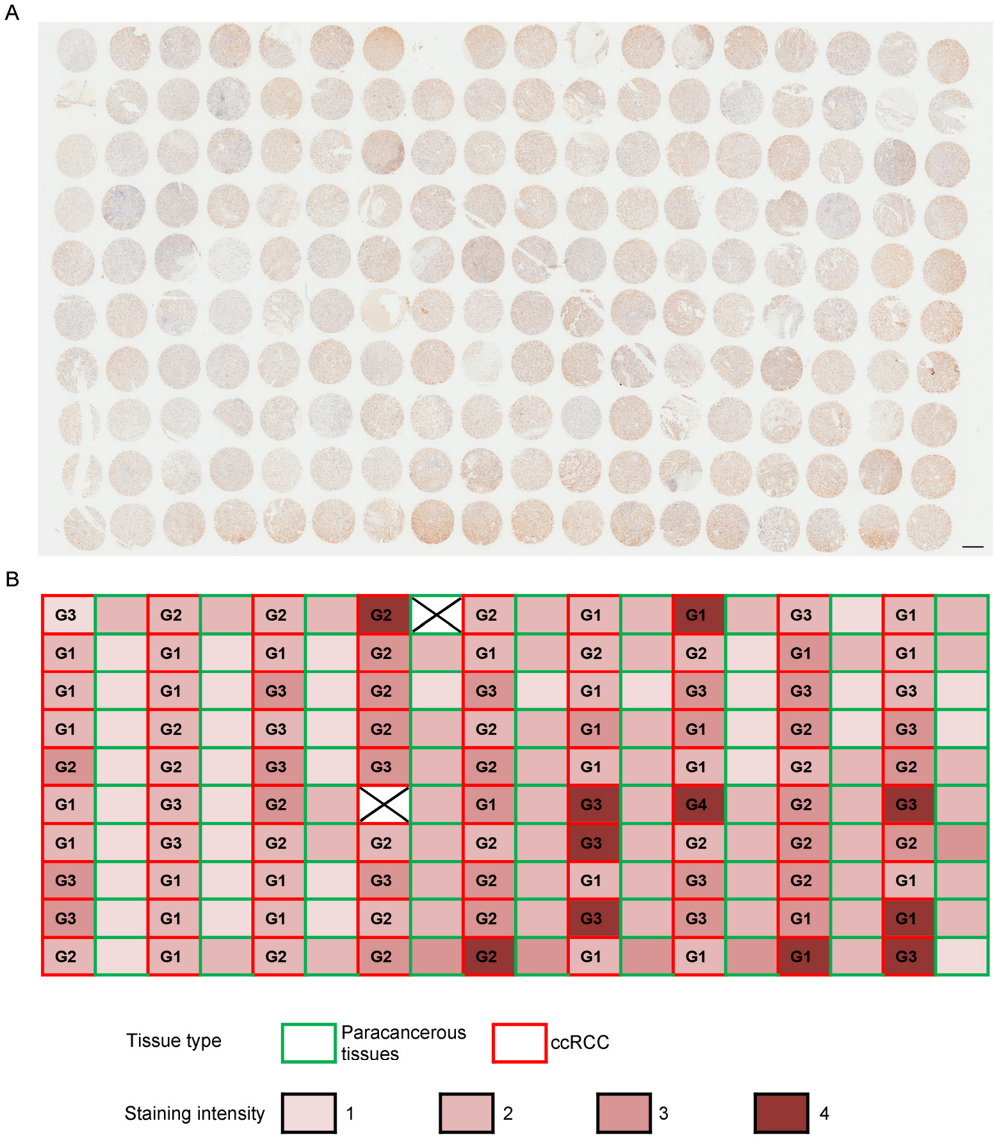

Supplementary Figure S4. Clinical information about the tissue microarray. (A) Overview of tissues microarray. Scale bar: 800 μm. **(B)** Pathological grade, tissue type and staining intensity of the tissue microarray.

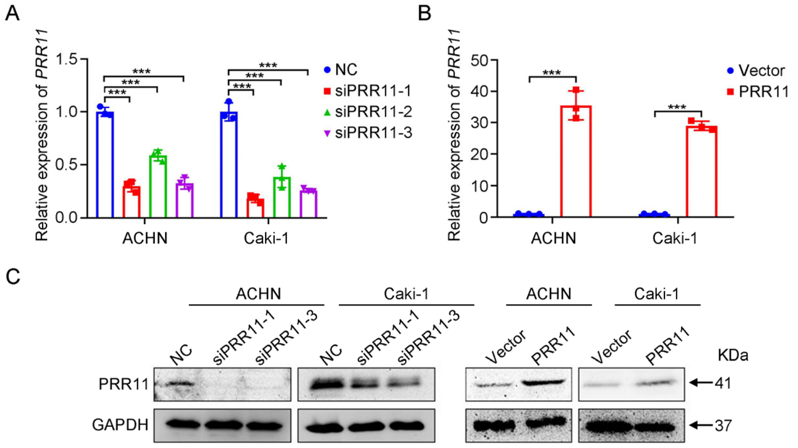

Supplementary Figure S5. The efficiency of *PRR11* knockdown and overexpression in ACHN and Caki-1 cells was verified. (A-C) qRT-PCR and Western blotting assays confirmed the efficiency of *PRR11* silencing and upregulation in ccRCC cells (ACHN and Caki-1 cells) (n = 3). The data are shown as the mean ± SD. One-way ANOVA test **(A)** and two-tailed *t*-test **(B)** analyses were performed. *** $p < 0.001$.

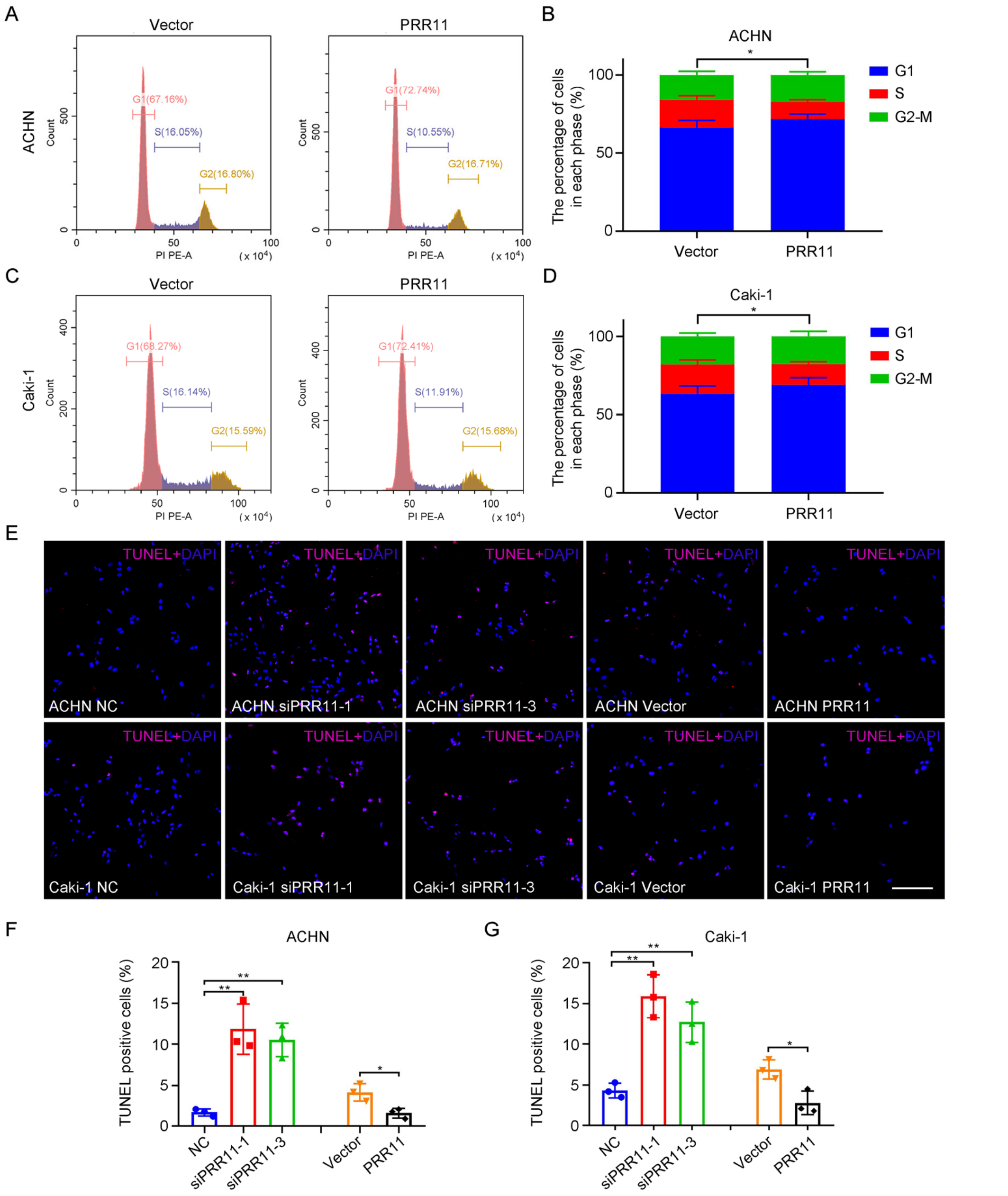

Supplementary Figure S6. The effect of PRR11 expression on ccRCC cells was evaluated by flow cytometry and TUNEL assay. (A-D) Flow cytometry analysis of the effect of PRR11 overexpression on the cell cycle progression of ccRCC (n = 3). **(E-G)** The TUNEL assay was used to evaluate the effect of PRR11 expression on ccRCC cell apoptosis (n = 3). Scale bar: 100 μm. The data are shown as the mean ± SD. Two-tailed *t*-test analyses **(B, D, F right, G right)** and one-way ANOVA test **(F left, G left)** analyses were performed. * $p < 0.05$, ** $p < 0.01$.

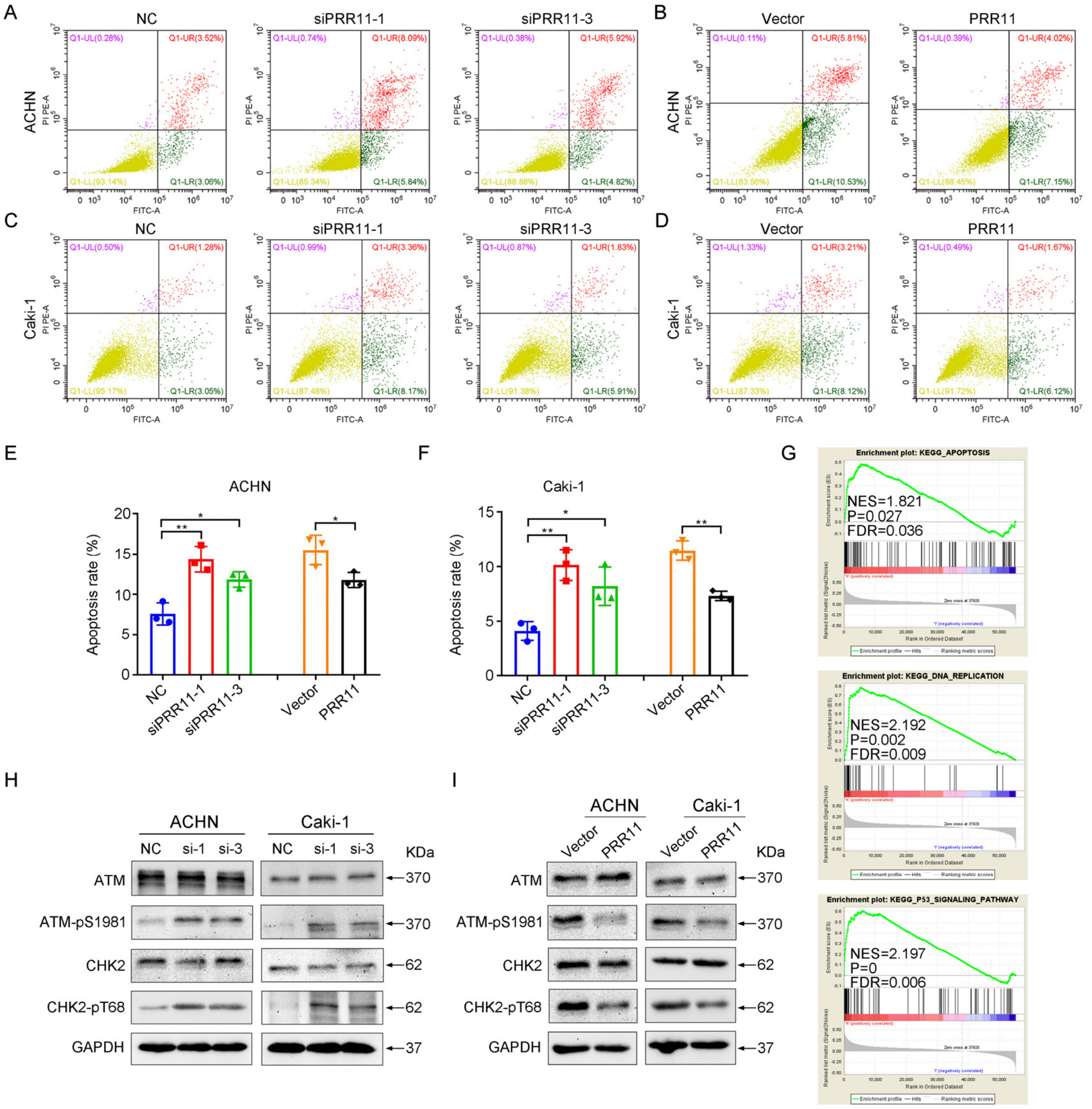

Supplementary Figure S7. The effect of PRR11 on the apoptosis of ccRCC cells was analyzed by flow cytometry. (A-F) *PRR11* silencing led to an increase in the percentage of apoptotic ccRCC cells, while overexpression of PRR11 led to a decrease in apoptosis (n = 3). **(G)** GSEA revealed the correlation between increased PRR11 expression and apoptosis, DNA replication and the p53 signaling pathway. NES, normalized enrichment score. Nominal p values calculated by the permutation test and FDR-corrected q values were obtained by multiple hypothesis tests in GSEA. **(H-I)** Expression of ATM/CHK2 pathway-related proteins was detected in *PRR11*-silenced or PRR11-overexpressing ccRCC cells by Western blotting analysis (n = 3). The data are shown as the mean ± SD. One-way ANOVA test **(E left, F left)** and two-tailed *t*-test **(E right, F right)** analyses were performed. * $p < 0.05$, ** $p < 0.01$.

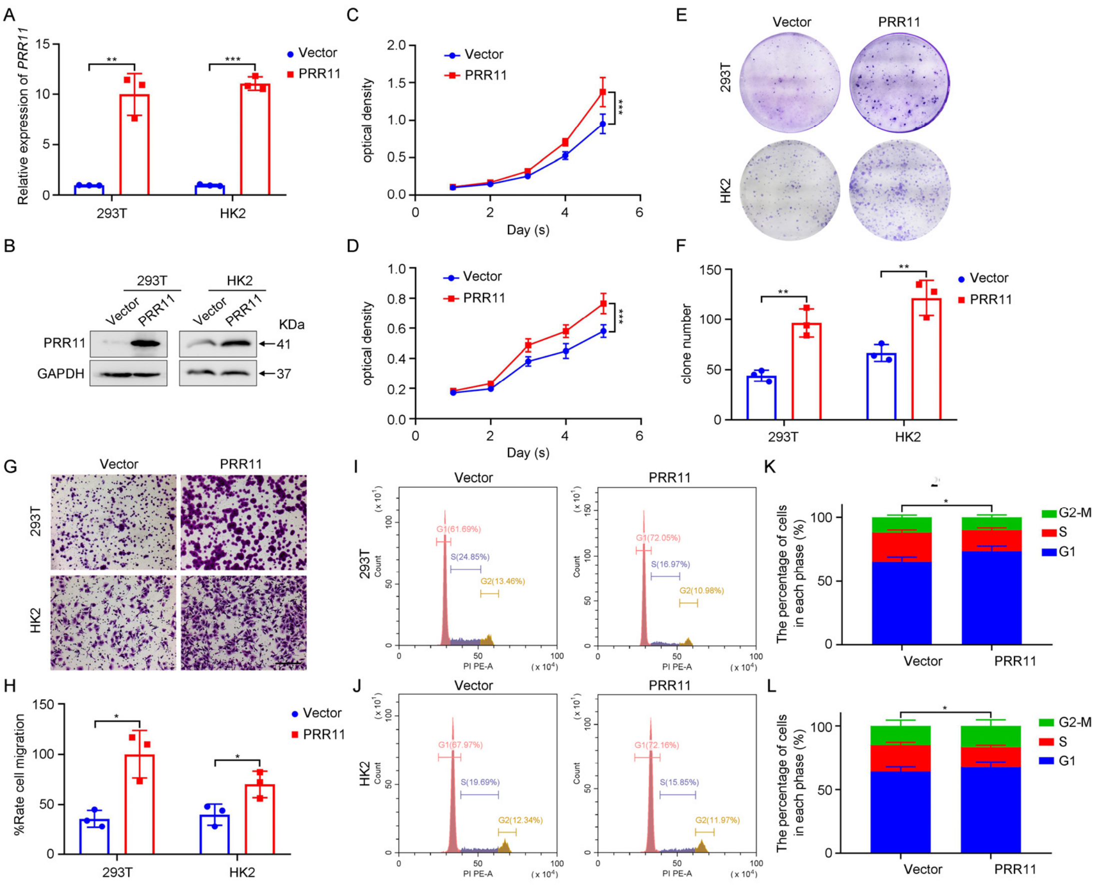

Supplementary Figure S8. The overexpression of PRR11 promoted the proliferation and migration of nonmalignant renal cells in vitro. (A-B) The overexpression efficiency of LV-PRR11 in 293T cells and HK2 cells was verified at the transcriptional and translational levels (n = 3). **(C-D)** MTT assay was used to evaluated the proliferation of 293T cells and HK2 cells overexpressing PRR11 (n = 3). **(E-F)** A clonogenic assay was used to assess the colony formation ability of PRR11-overexpressing 293T cells and HK2 cells, and the clone numbers were statistically analyzed (n = 3). **(G-H)** A Transwell migration assay was used to evaluate the migration ability of PRR11-overexpressing 293T cells and HK2 cells, and the relative number of migrated cells was statistically analyzed (n = 3). Scale bar: 150 μm. **(I-L)** Flow cytometry analysis of the effect of PRR11 overexpression on the cell cycle progression of 293T cells and HK2 cells (n = 3). The data are shown as the mean ± SD. Two-tailed *t*-test analyses were performed. * $p < 0.05$, ** $p < 0.01$, *** $p < 0.001$.

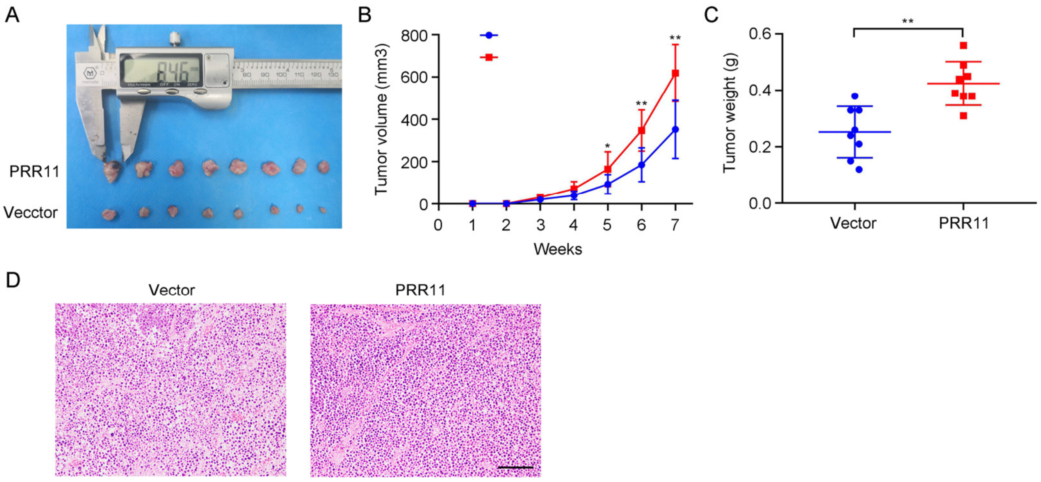

Supplementary Figure S9. PRR11 promoted tumorigenesis in vivo. (A) LV-Vector and LV-PRR11 293T cells were subcutaneously injected into nude mice to establish xenograft models. After 7 weeks, the xenografts were removed and imaged (n = 8). **(B-C)** Analysis and evaluation of the xenograft volumes and weights of LV-Vector group (blue dots) and LV-PRR11 group (red squares). **(D)** HE staining was used to determine the degree of tumor malignancy. Scale bar: 200 μm. The data are shown as the mean ± SD. Two-tailed *t*-test analyses were performed. * $p < 0.05$, ** $p < 0.01$.

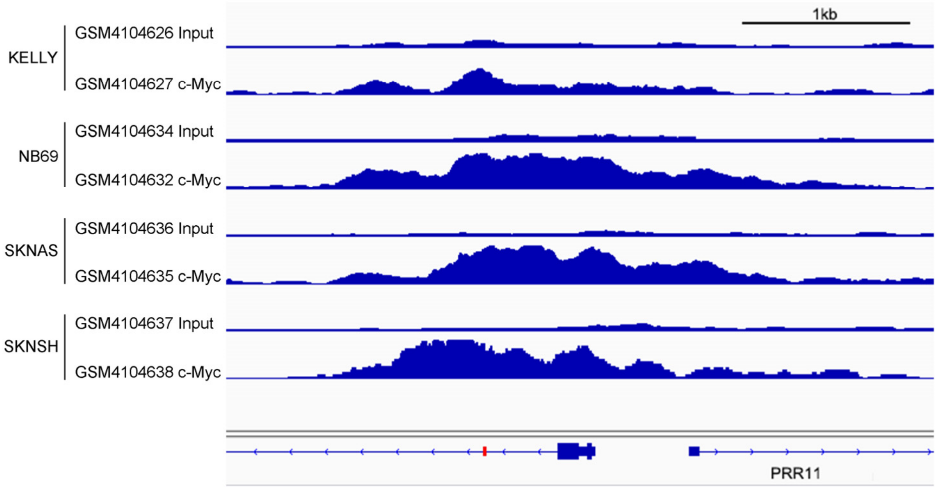

Supplementary Figure S10. Browser view of the highly conserved ChIP-Seq peak on PRR11. The binding of c-Myc to the PRR11 promoter region was confirmed by c-Myc ChIP-Seq data from the GSE138295 dataset in the GEO public database.

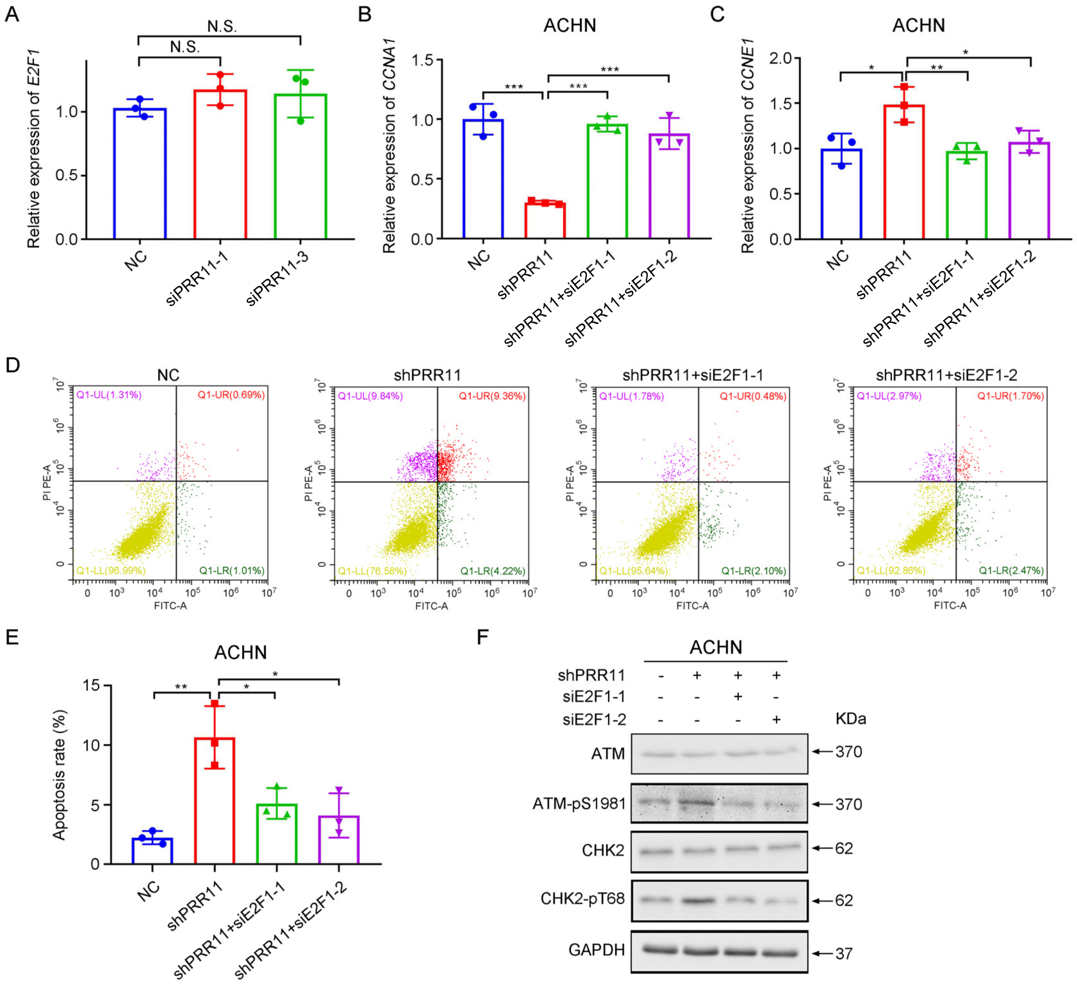

Supplementary Figure S11. Expression of related genes and downregulation of *E2F1* expression reversed the shPRR11-mediated promotion of apoptosis in ACHN cells. (A) qRT-PCR analysis of the effect of *PRR11* silencing on the mRNA expression of *E2F1* in ACHN cells (n = 3). **(B-C)** qRT-PCR analysis of the effects on *CCNA1* and *CCNE1* mRNA expression in rescue experiments with siE2F1 (n = 3). **(D-E)** Flow cytometry analysis showed that *E2F1* knockdown could significantly reverse the apoptotic effect of shPRR11 transfection in ACHN cells (n = 3). **(F)** The expression of apoptosis-related genes in ccRCC cells was detected by western blotting (n = 3). The data are shown as the mean ± SD. One-way ANOVA test **(A)** and two-way ANOVA test **(B-C, E)** analyses were performed. N.S. = No significance, * $p < 0.05$, ** $p < 0.01$, *** $p < 0.001$.

Supplementary Table S1. Cox analysis of 248 ccRCC patients based on TCGA database.

**Kidney cancer patenits** (n=248)	Univariate analysis			Multivariate analysis		
	HR	95%CI	*P* value	HR	95%CI	*P* value
***PRR11* expression** (high/low)	2.081	1.521-2.846	<0.001	1.532	1.053-2.229	0.002
**Age**	1.023	1.005-1.041	0.012	1.032	1.012-1.053	0.717
**Gender** (male/female)	1.013	0.666-1.541	0.951	1.088	0.688-1.722	0.066
**Grade** (G2-G4 VS. G1)	2.242	1.682-2.988	<0.001	1.376	0.980-1.934	0.375
**Stage** (S2-S4 VS. S1)	1.862	1.541-2.251	<0.001	1.267	0.751-2.139	0.736
**T Stage** (T2-T4 VS. T1)	1.943	1.538-2.456	<0.001	1.088	0.666-1.776	0.079
**M Stage** (M1 VS. M0)	4.073	2.634-6.300	<0.001	2.073	0.919-4.677	0.542
**N Stage** (N1 VS. N0)	2.932	1.521-2.846	0.001	1.262	0.598-2.663	0.026

Supplementary Table S2. List of primers for qRT-PCR.

Gene name	Forward primer (5'-3')	Reverse primer (5'-3')
PRR11	AAAGATGGACCCATGCAGATAAC	TGCTTTCGGCGATGGTATAAG
GAPDH	TGCACCACCAACTGCTTAG	GATGCAGGGATGATGTTC
c-Myc	CGTCCTCGGATTCTCTGCTC	GATTTCTTCCTCATCTTCTTGTTC
CCNA1	GAGGTCCCGATGCTTGTCAG	GTTAGCAGCCCTAGCACTGTC
CCNE1	AAGGAGCGGGACACCATGA	ACGGTCACGTTTGCCTTCC
VHL	GCAGGCGTCGAAGAGTACG	CGGACTGCGATTGCAGAAGA

Supplementary Table S3. List of antibodies.

**Antigens**	**Species antibodies raised in**	**Dilution**	**Supplier**
PRR11	Rabbit, polyclonal	1:1,000 (WB)<br>1:100 (IHC)	Cusabio, Cat. #PA836225LA01HU
GAPDH	Mouse, monoclonal	1:2,000 (WB)	Santa Cruz Biotechnology Inc., USA, Cat. #sc-365062
E2F1	Rabbit, monoclonal	1:1,000 (WB)<br>1:50 (IP)	Abcam, UK, Cat. #ab179445
c-Myc	Rabbit, monoclonal	1:1,000 (WB)	Abcam, UK, Cat. #ab32072
p21	Rabbit, monoclonal	1:1,000 (WB)	Abcam, UK, Cat. #ab109520
CDK2	Rabbit, monoclonal	1:1,000 (WB)	Abcam, UK, Cat. #ab32147
CDK4	Rabbit, monoclonal	1:1,000 (WB)	Abcam, UK, Cat. #ab108357
CDK6	Rabbit, monoclonal	1:1,000 (WB)	Abcam, UK, Cat. #ab124821
CCNA1	Rabbit, monoclonal	1:1,000 (WB)	Abcam, UK, Cat. #ab185619
CCNE1	Rabbit, monoclonal	1:1,000 (WB)	Abcam, UK, Cat. #ab33911
E-cadherin	Rabbit, monoclonal	1:500 (WB)	Cell Signaling Technology, USA, Cat. #3195
N-cadherin	Rabbit, monoclonal	1:500 (WB)	Cell Signaling Technology, USA, Cat. #13116
β-Catenin	Rabbit, monoclonal	1:1,000 (WB)	Cell Signaling Technology, USA, Cat. #8480
MMP9	Rabbit, monoclonal	1:1,000 (WB)	Cell Signaling Technology, USA, Cat. #13667
Vimentin	Rabbit, monoclonal	1:1,000 (WB)	Cell Signaling Technology, USA, Cat. #5741
Snail	Rabbit, monoclonal	1:1,000 (WB)	Cell Signaling Technology, USA, Cat. # 3879
Slug	Rabbit, monoclonal	1:1,000 (WB)	Cell Signaling Technology, USA, Cat. #9585
ATM	Rabbit, monoclonal	1:1,000 (WB)	Abcam, UK, Cat. #ab32420
CHK2	Rabbit, monoclonal	1:5,000 (WB)	Abcam, UK, Cat. #ab109413
ATM (pS1981)	Rabbit, monoclonal	1:10,000 (WB)	Abcam, UK, Cat. #ab81292
CHK2 (pT68)	Rabbit, monoclonal	1:2,000 (WB)	Abcam, UK, Cat. #ab32148
VHL	Rabbit, polyclonal	1:1,000 (WB)	Cell Signaling Technology, USA, Cat. #68547
c-Myc	Rabbit, polyclonal	1:50 (ChIP)	Cell Signaling Technology, USA, Cat. #9402
Ki-67	Mouse, monoclonal	1:200 (IHC)	Cell Signaling Technology, USA, Cat. #9449
Flag tag	Mouse, monoclonal	1:100 (IP)	Sigma, Cat. #F1804
HA tag	Mouse, monoclonal	1:100 (IP)	OriGene, Cat. #TA180128
Anti-Mouse-IgG (H+L)-HRP	Goat	1:10,000 (WB)	Jackson ImmunoResearch Inc, USA, Cat. #115-005-003
Anti-Rabbit-IgG (H+L)-HRP	Goat	1:10,000 (WB)	Jackson ImmunoResearch Inc, USA, Cat. #111-005-003

Supplementary Table S4. List of primers for PRR11 promoter.

Primer number	Forward primer (5’-3’)	Reverse primer (5’-3’)
Primer 1	TCAGCAACAAAGGTTGTGGT	CATCTGAAAGCAGGATGCAC
Primer 2	CCCGAGTAGCTGGGACTACA	TTGAGACTACCCTGGCCAAC
Primer 3	GGATGCCTTTCCTGTCTCAA	GTGAGGGAAGAGGTGTGAGC
Primer 4	CATGGCAAAATCCCATCTCT	ATCCTGCTTCAGCCTCTTGA

Full unedited gel for Figure 1

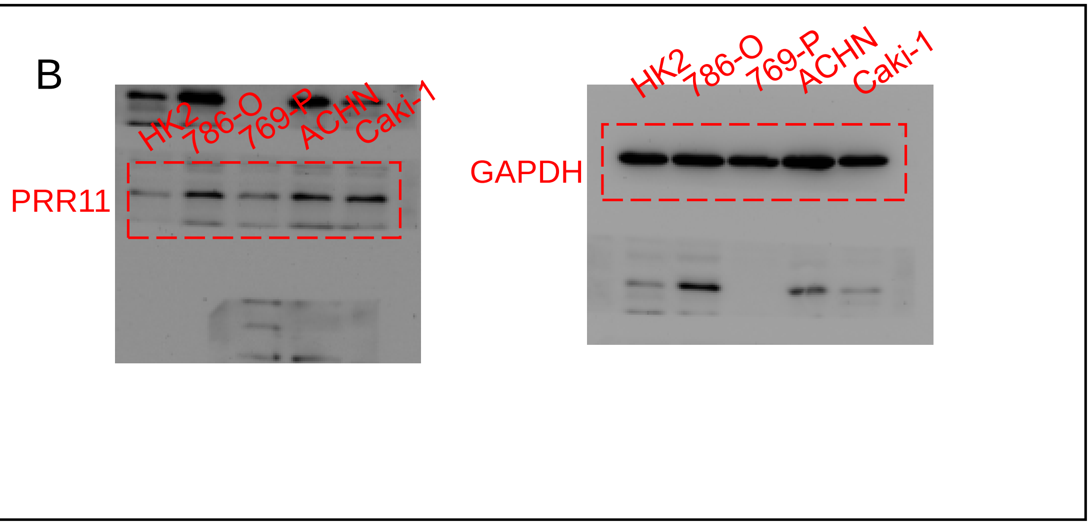

Full unedited gel for Figure 3

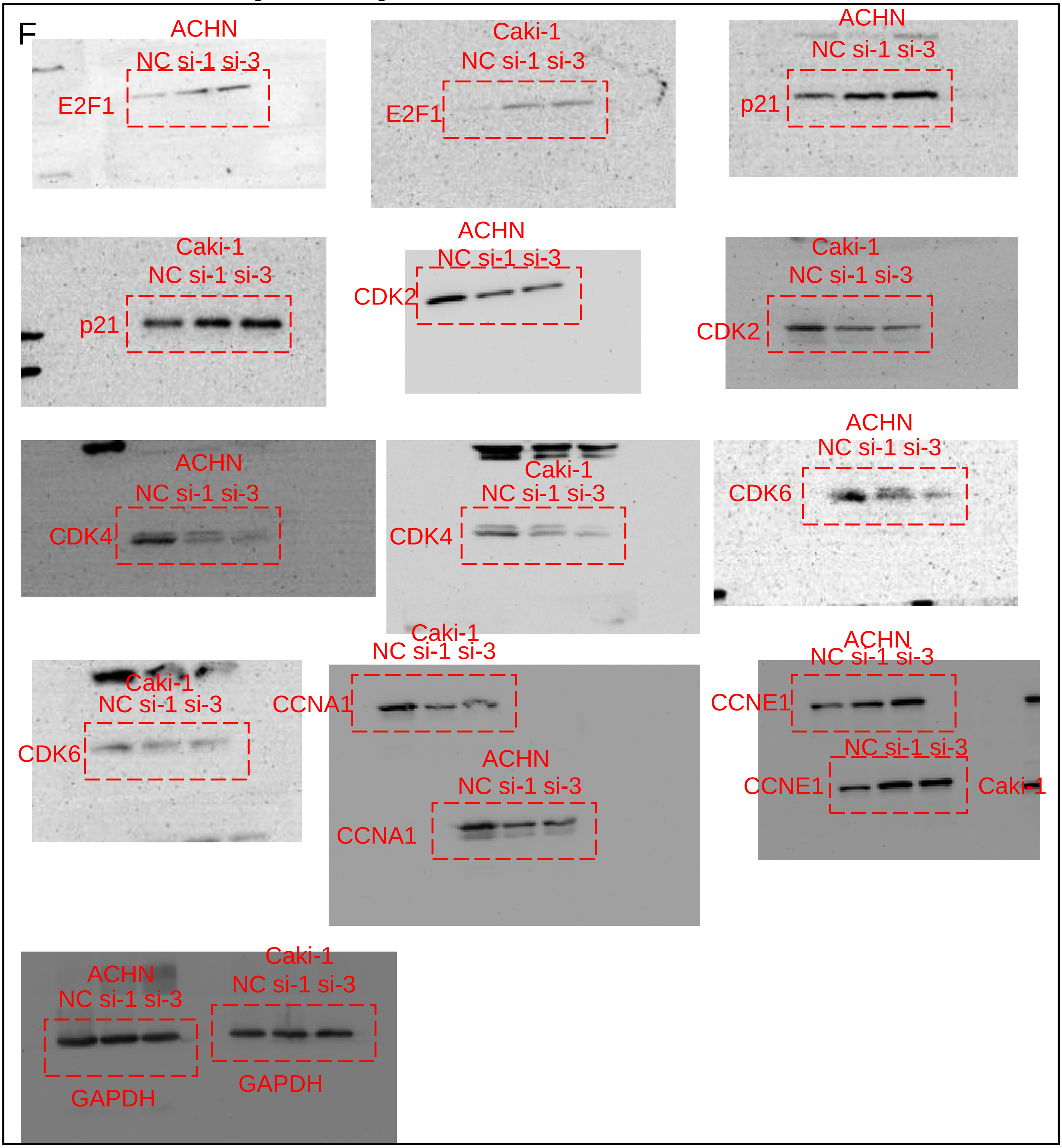

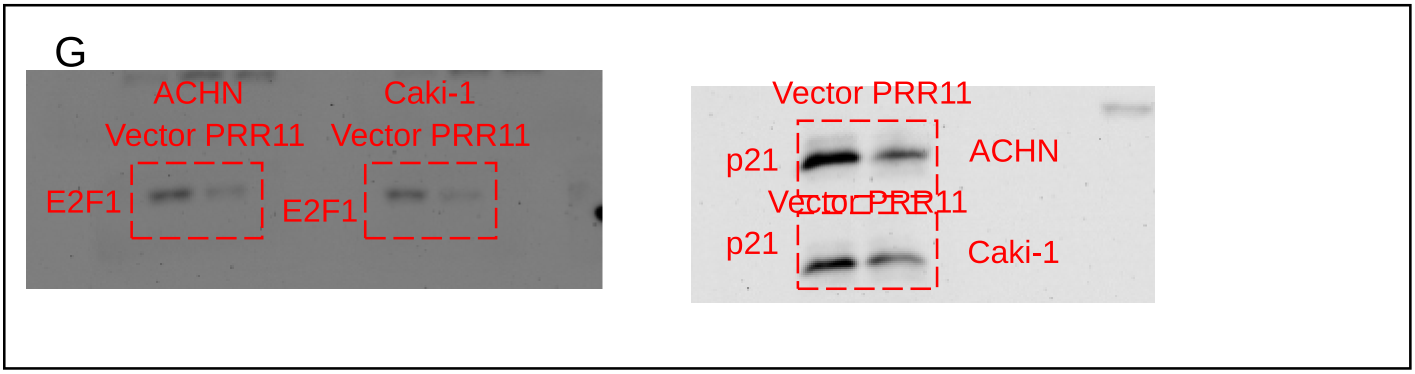

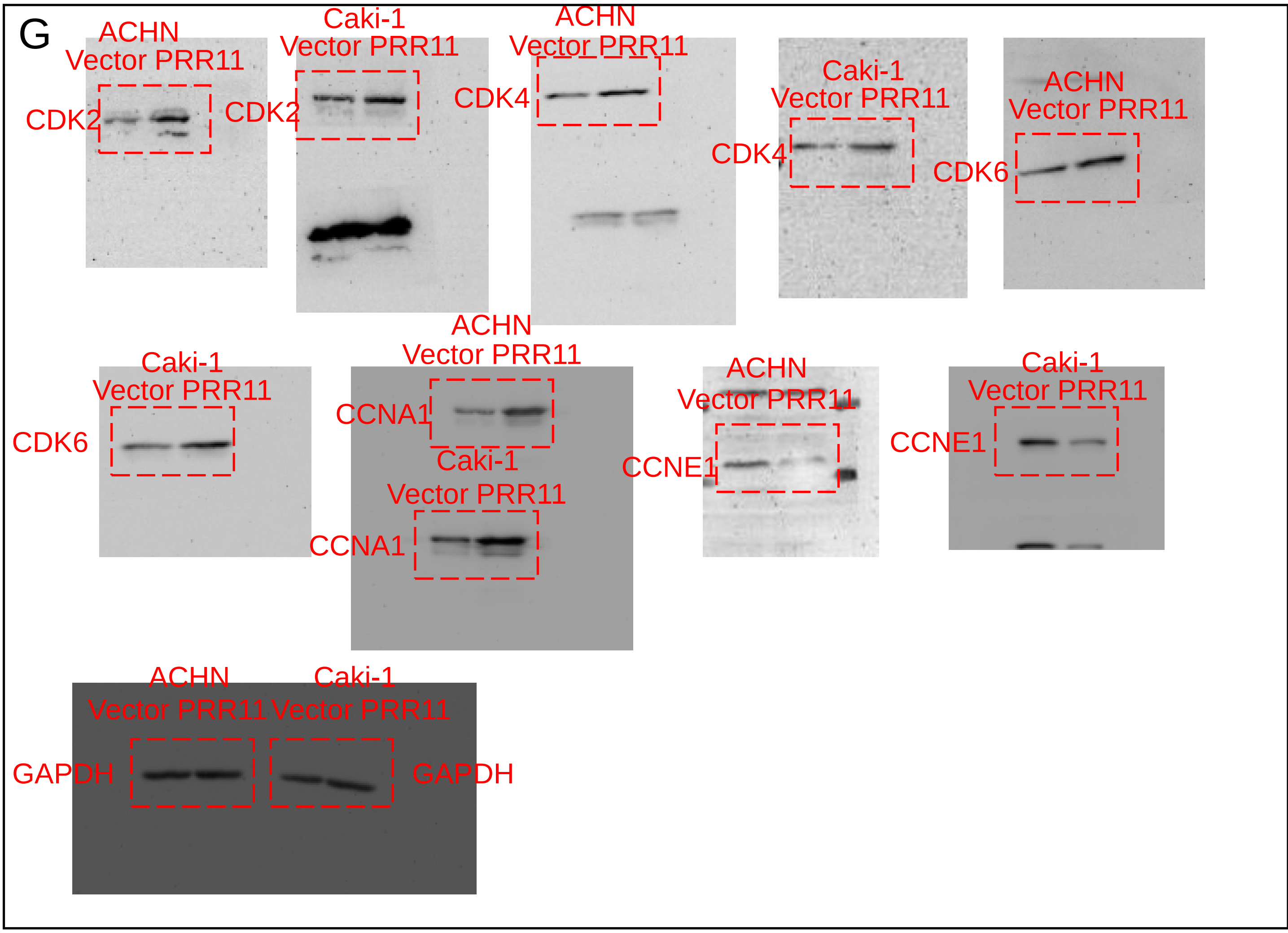
G
ACHN
Vector PRR11
CDK2
Caki-1
Vector PRR11
CDK2
ACHN
Vector PRR11
CDK4
Caki-1
Vector PRR11
CDK4
ACHN
Vector PRR11
CDK6
Caki-1
Vector PRR11
CDK6
ACHN
Vector PRR11
CCNA1
Caki-1
Vector PRR11
CCNA1
ACHN
Vector PRR11
CCNE1
Caki-1
Vector PRR11
CCNE1
ACHN
Vector PRR11
Caki-1
Vector PRR11
GAPDH
GAPDH

Full unedited gel for Figure 4

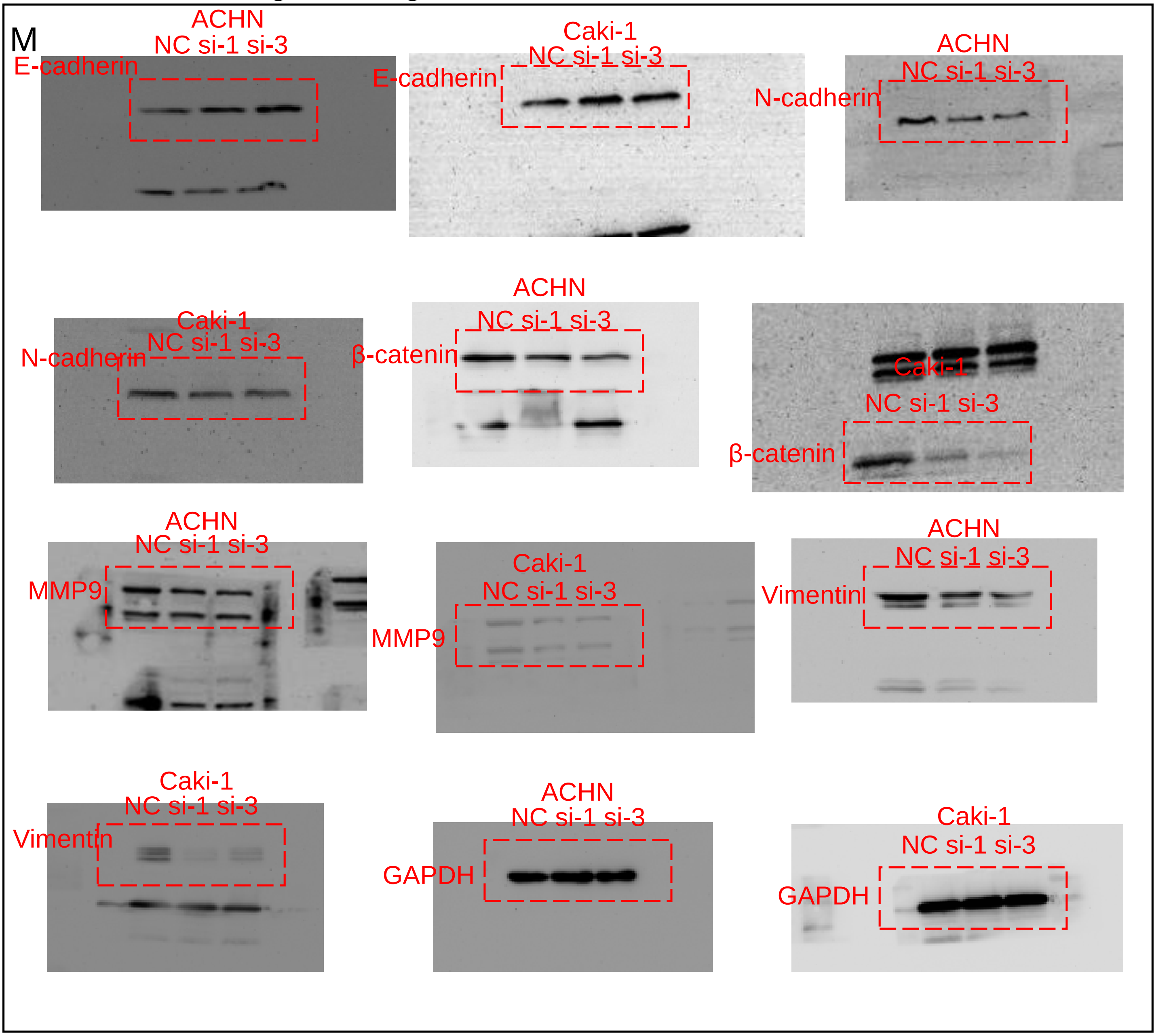

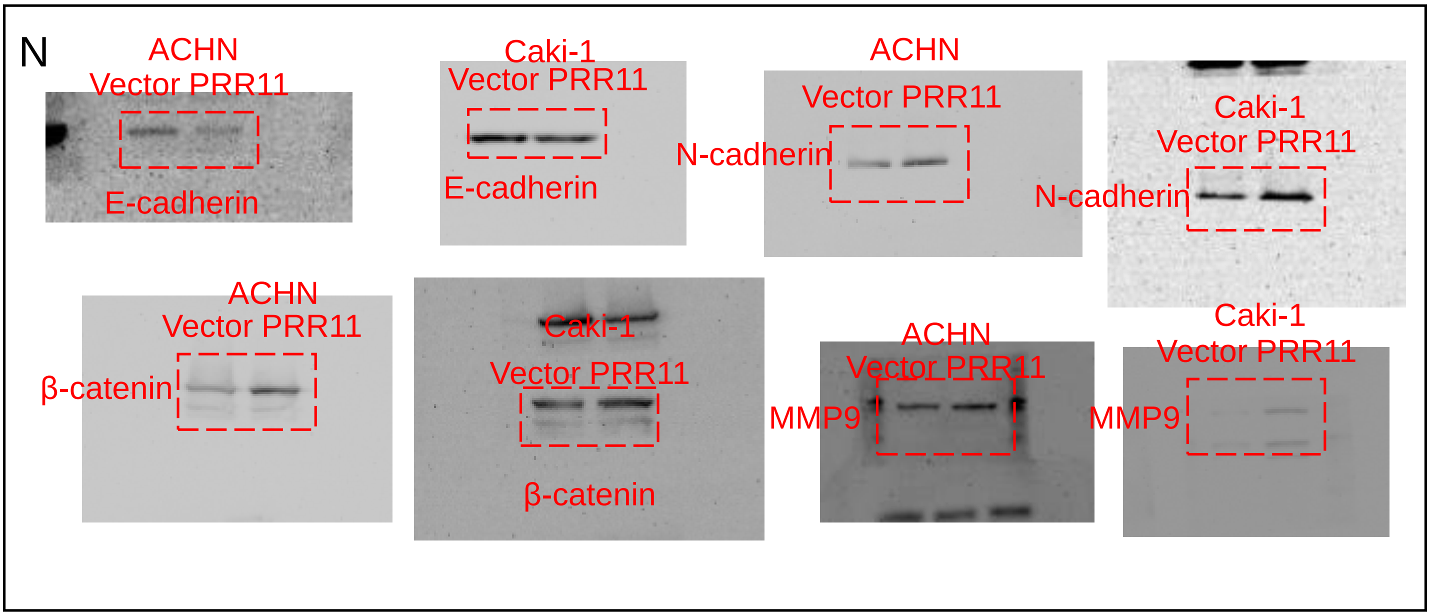

N

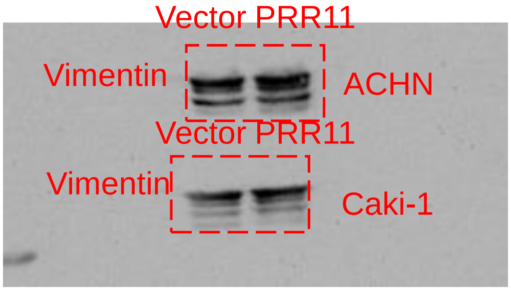
Vector PRR11
Vimentin
ACHN
Vector PRR11
Vimentin
Caki-1

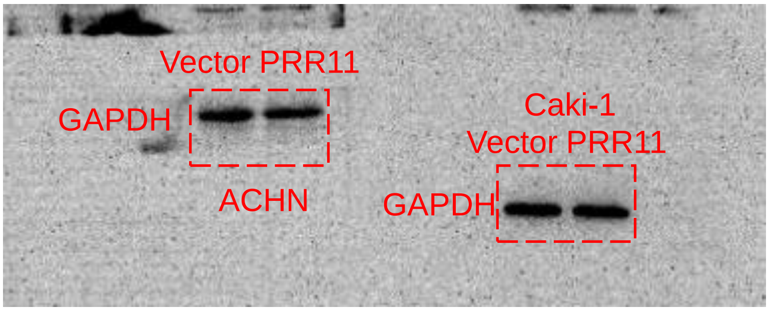
Vector PRR11
GAPDH
ACHN
Caki-1
Vector PRR11
GAPDH

Full unedited gel for Figure 5

B

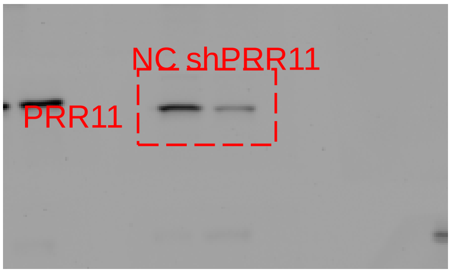

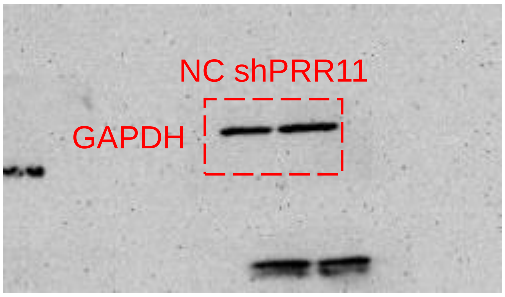

Full unedited gel for Figure 6

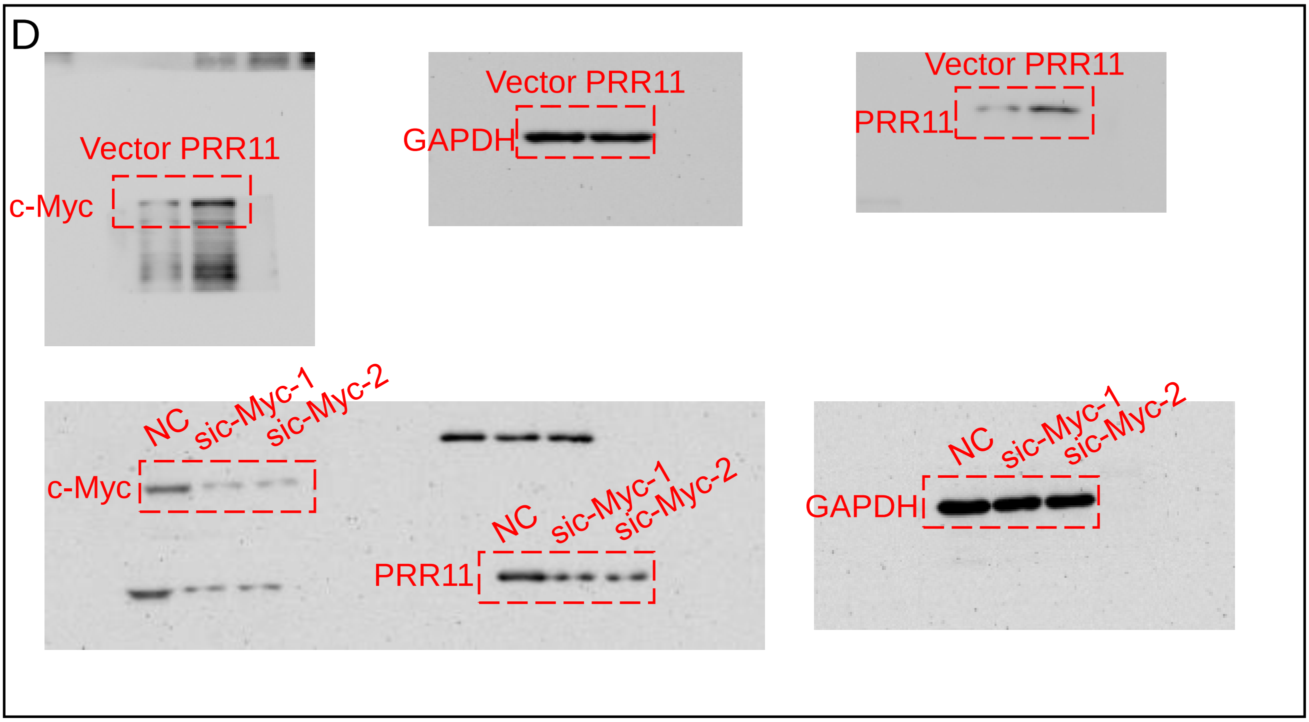

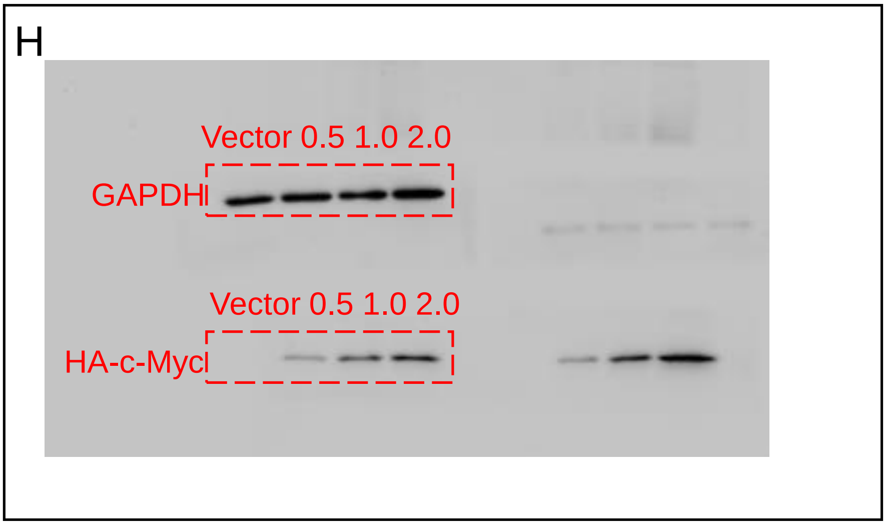

Full unedited gel for Figure 7

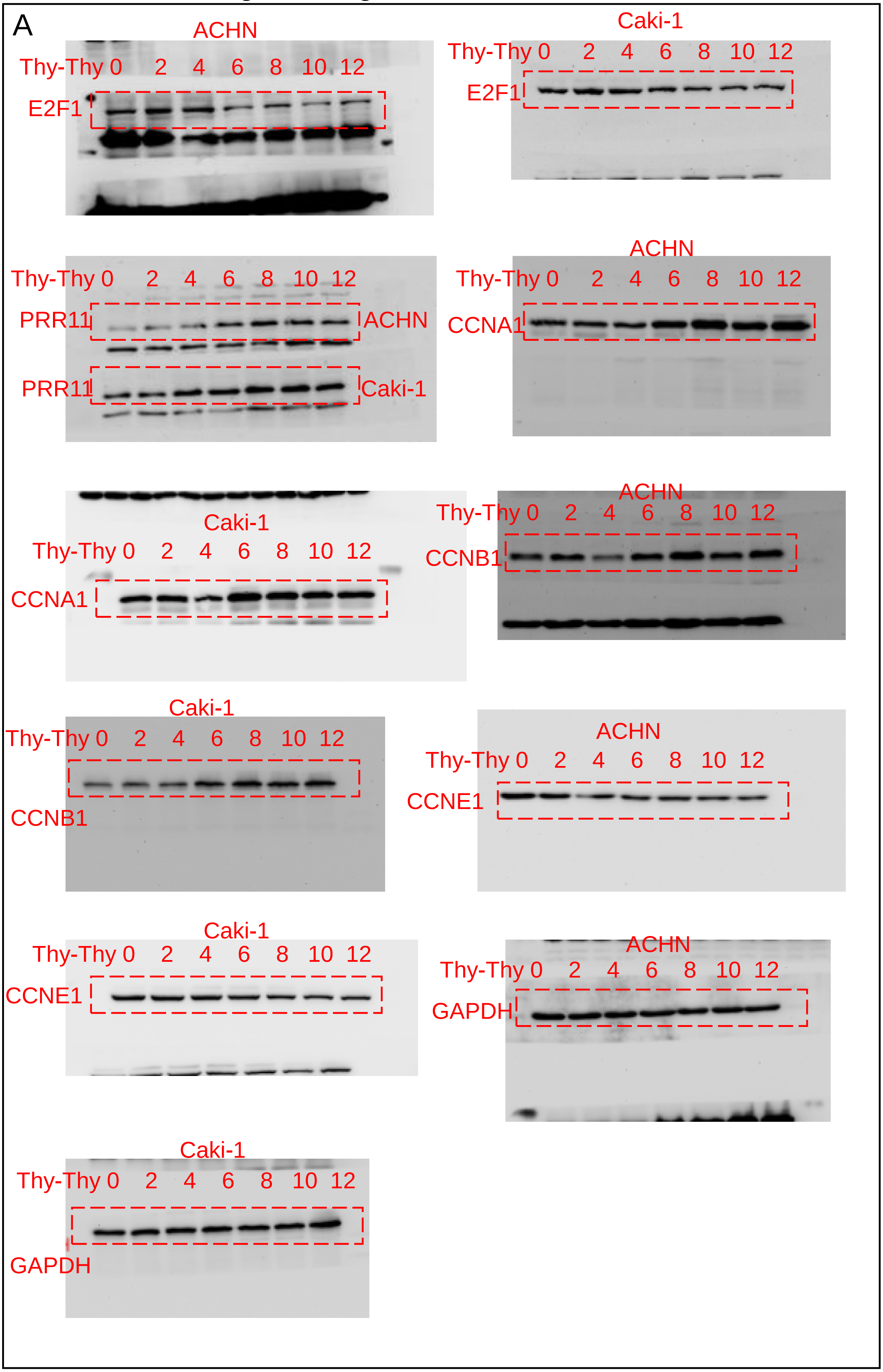

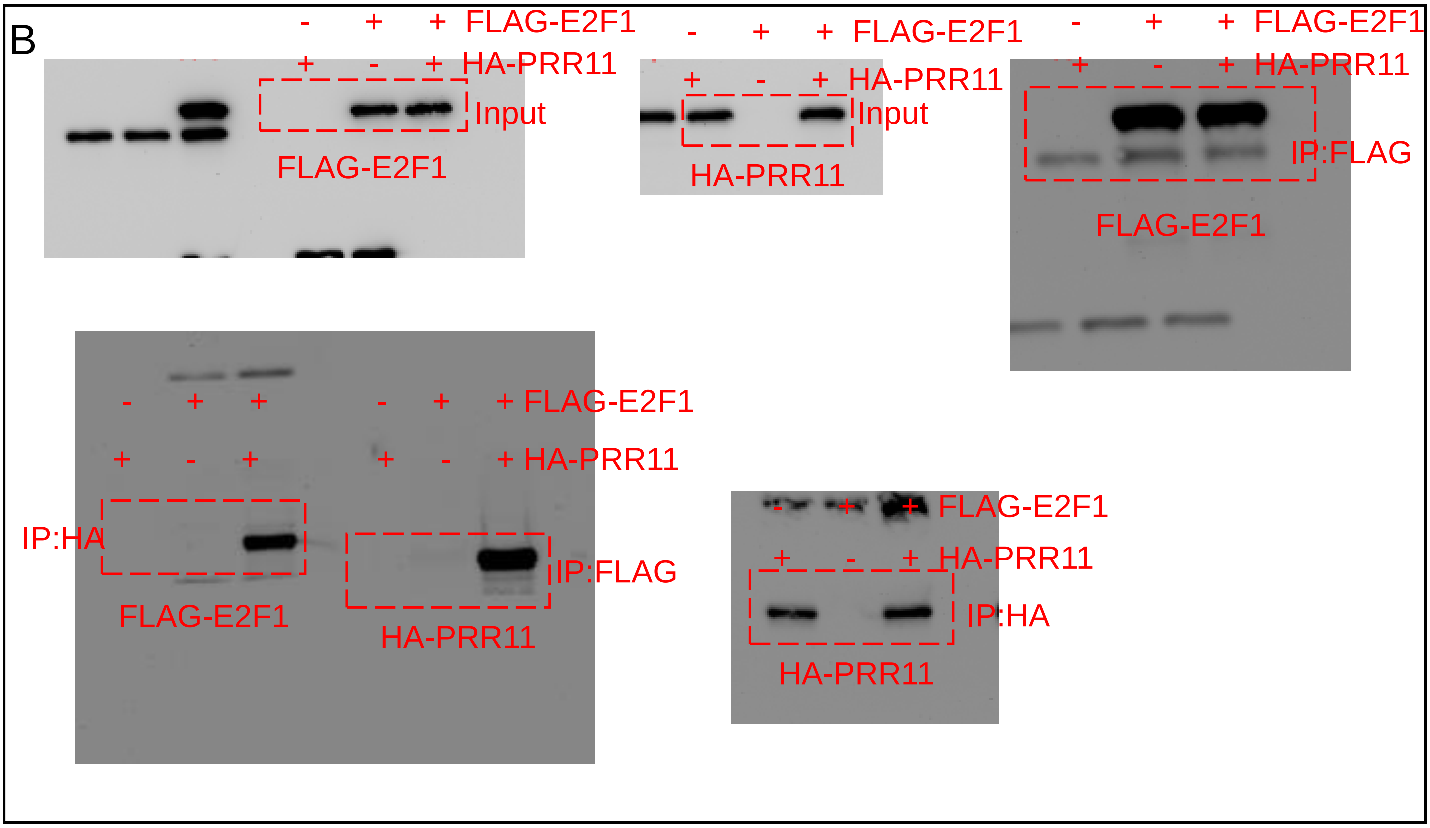
B
- + + FLAG-E2F1
+ - + HA-PRR11
Input
FLAG-E2F1
- + + FLAG-E2F1
+ - + HA-PRR11
Input
HA-PRR11
- + + FLAG-E2F1
+ - + HA-PRR11
IP:FLAG
FLAG-E2F1
- + + FLAG-E2F1
+ - + HA-PRR11
IP:HA
FLAG-E2F1
- + + FLAG-E2F1
+ - + HA-PRR11
IP:FLAG
HA-PRR11
- + + FLAG-E2F1
+ - + HA-PRR11
IP:HA
HA-PRR11

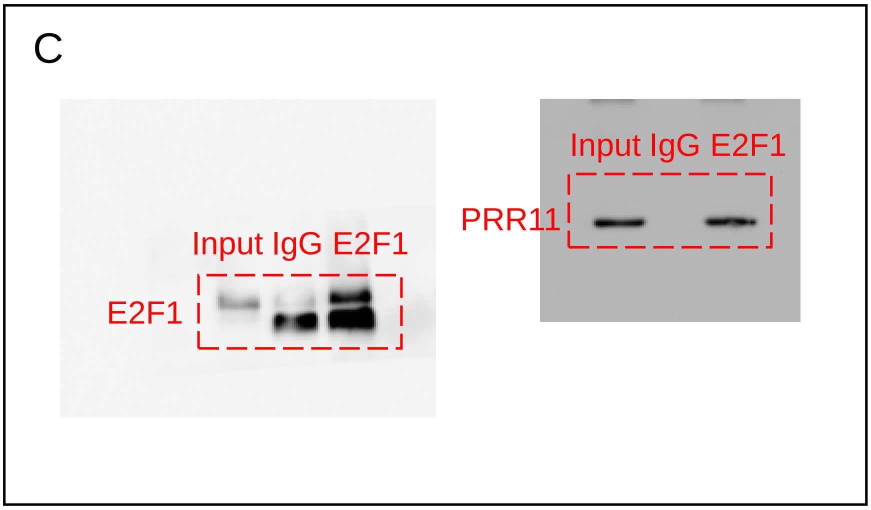
C
Input IgG E2F1
E2F1
Input IgG E2F1
PRR11

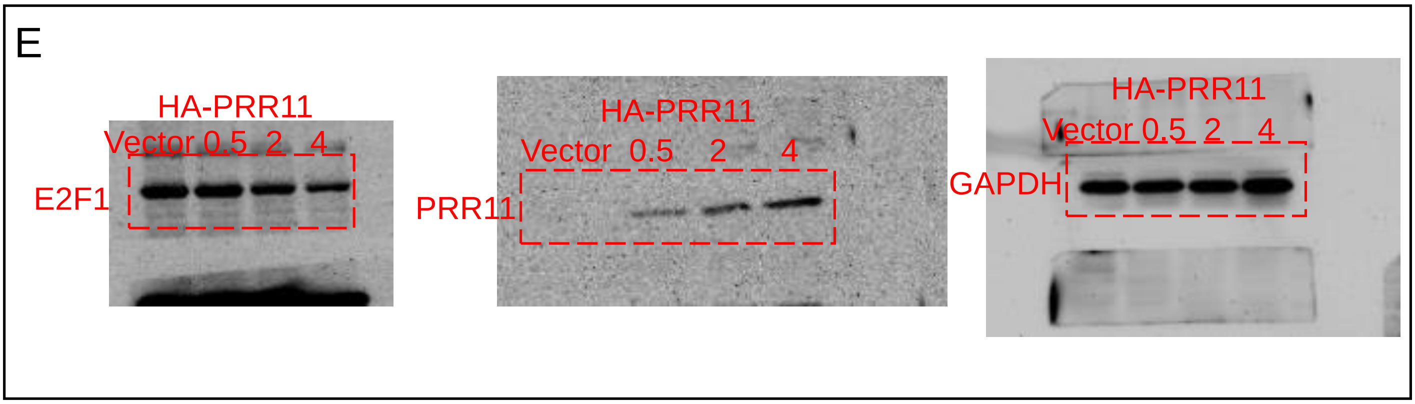
E
HA-PRR11
Vector 0.5 2 4
E2F1
HA-PRR11
Vector 0.5 2 4
PRR11
HA-PRR11
Vector 0.5 2 4
GAPDH

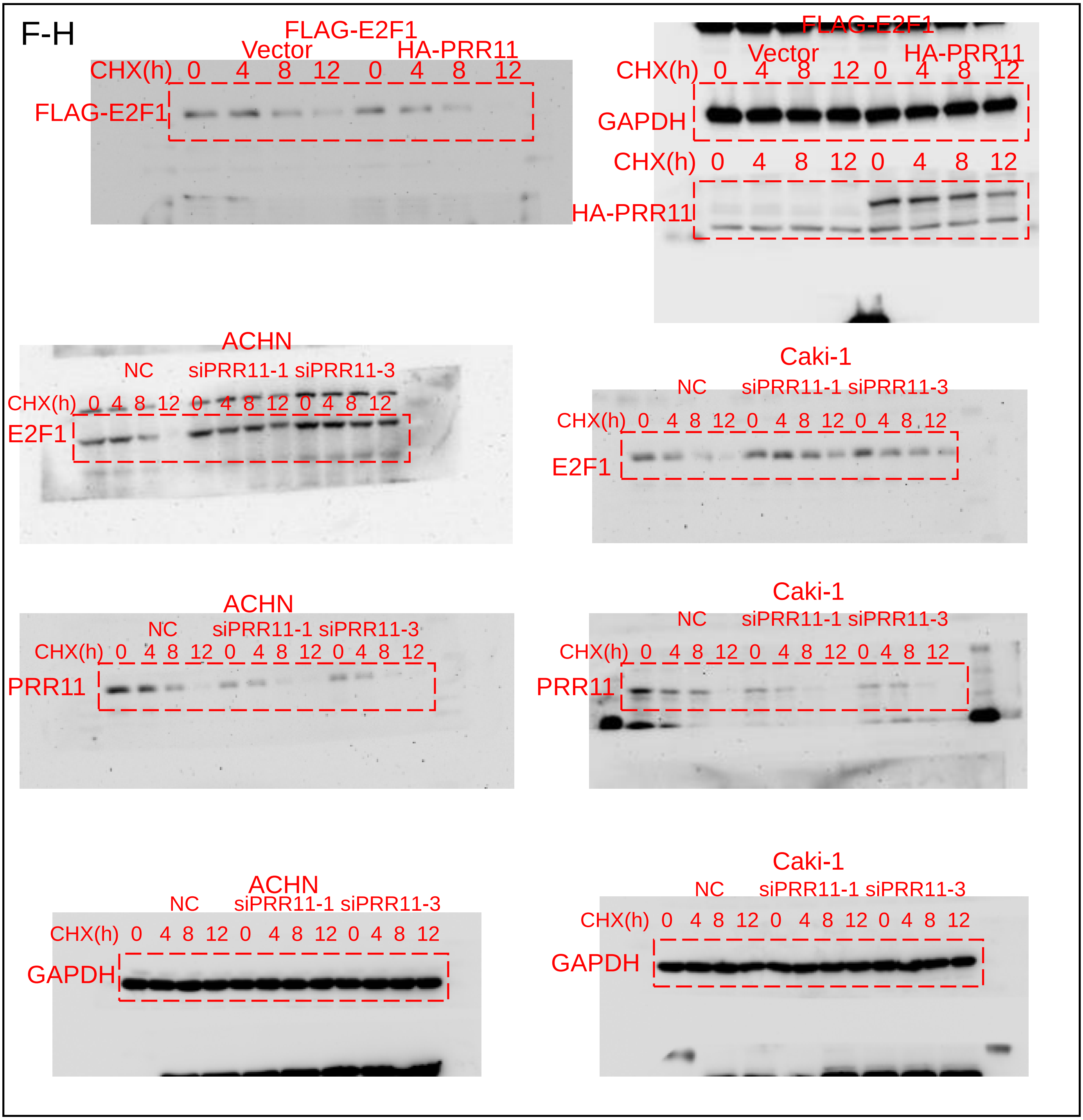
F-H
FLAG-E2F1
Vector
HA-PRR11
CHX(h) 0 4 8 12 0 4 8 12
FLAG-E2F1
FLAG-E2F1
Vector
HA-PRR11
CHX(h) 0 4 8 12 0 4 8 12
GAPDH
CHX(h) 0 4 8 12 0 4 8 12
HA-PRR11
ACHN
NC siPRR11-1 siPRR11-3
CHX(h) 0 4 8 12 0 4 8 12 0 4 8 12
E2F1
Caki-1
NC siPRR11-1 siPRR11-3
CHX(h) 0 4 8 12 0 4 8 12 0 4 8 12
E2F1
ACHN
NC siPRR11-1 siPRR11-3
CHX(h) 0 4 8 12 0 4 8 12 0 4 8 12
PRR11
Caki-1
NC siPRR11-1 siPRR11-3
CHX(h) 0 4 8 12 0 4 8 12 0 4 8 12
PRR11
ACHN
NC siPRR11-1 siPRR11-3
CHX(h) 0 4 8 12 0 4 8 12 0 4 8 12
GAPDH
Caki-1
NC siPRR11-1 siPRR11-3
CHX(h) 0 4 8 12 0 4 8 12 0 4 8 12
GAPDH

Full unedited gel for Figure 8

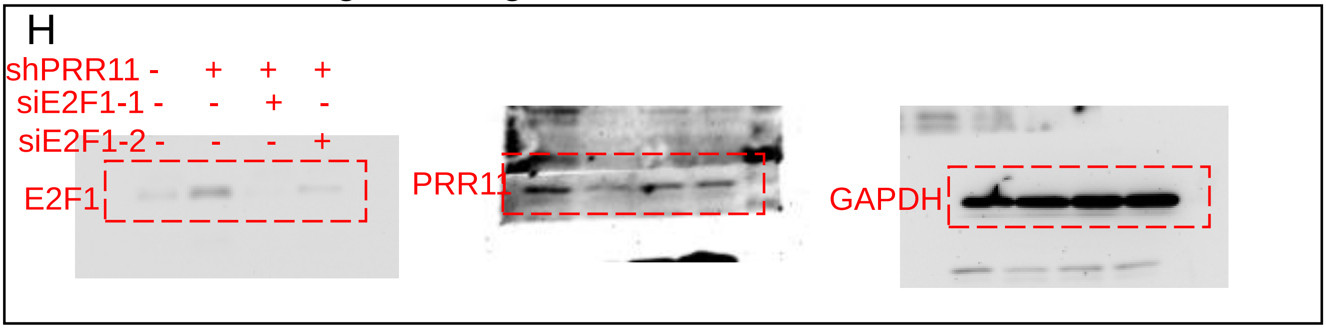

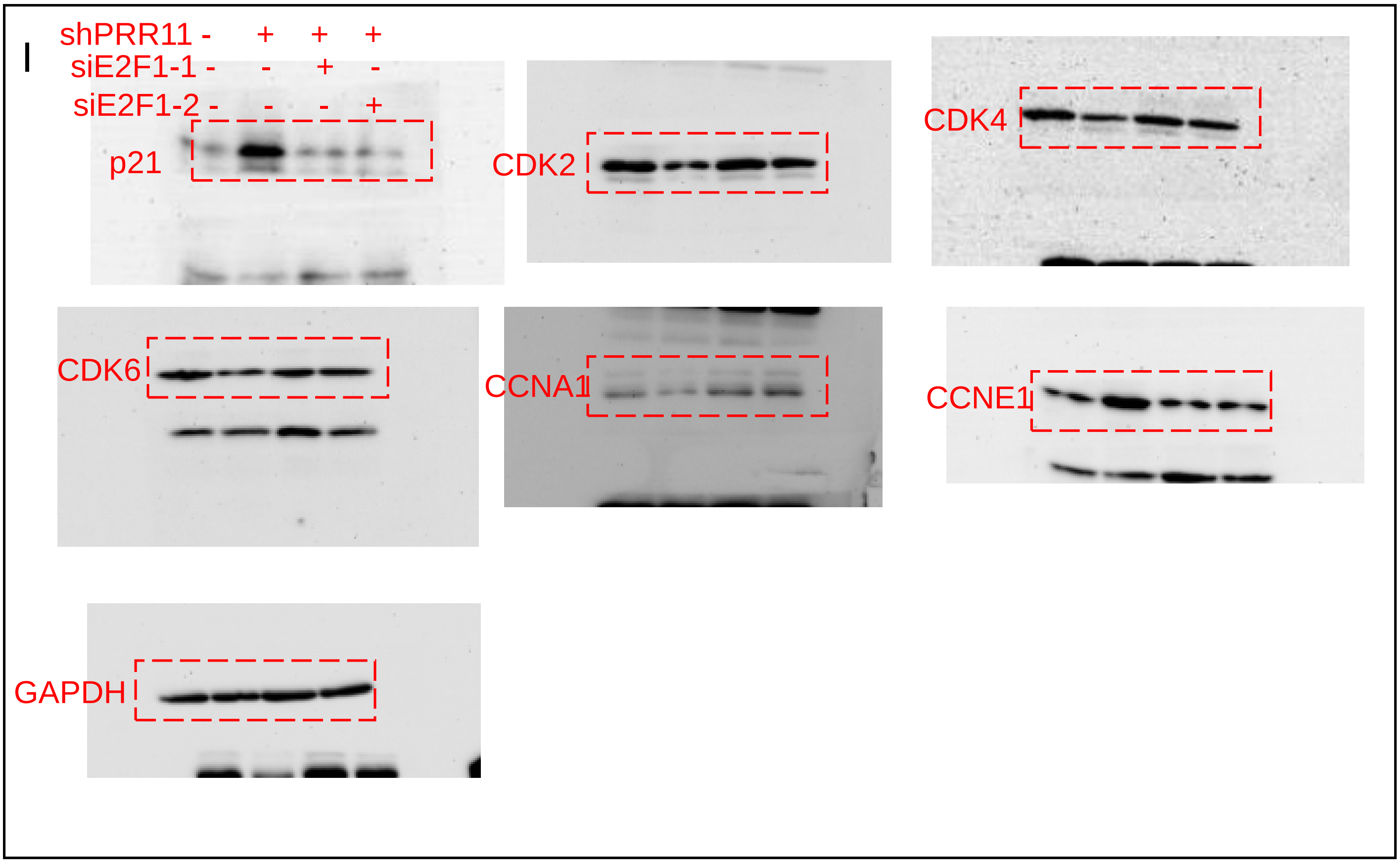

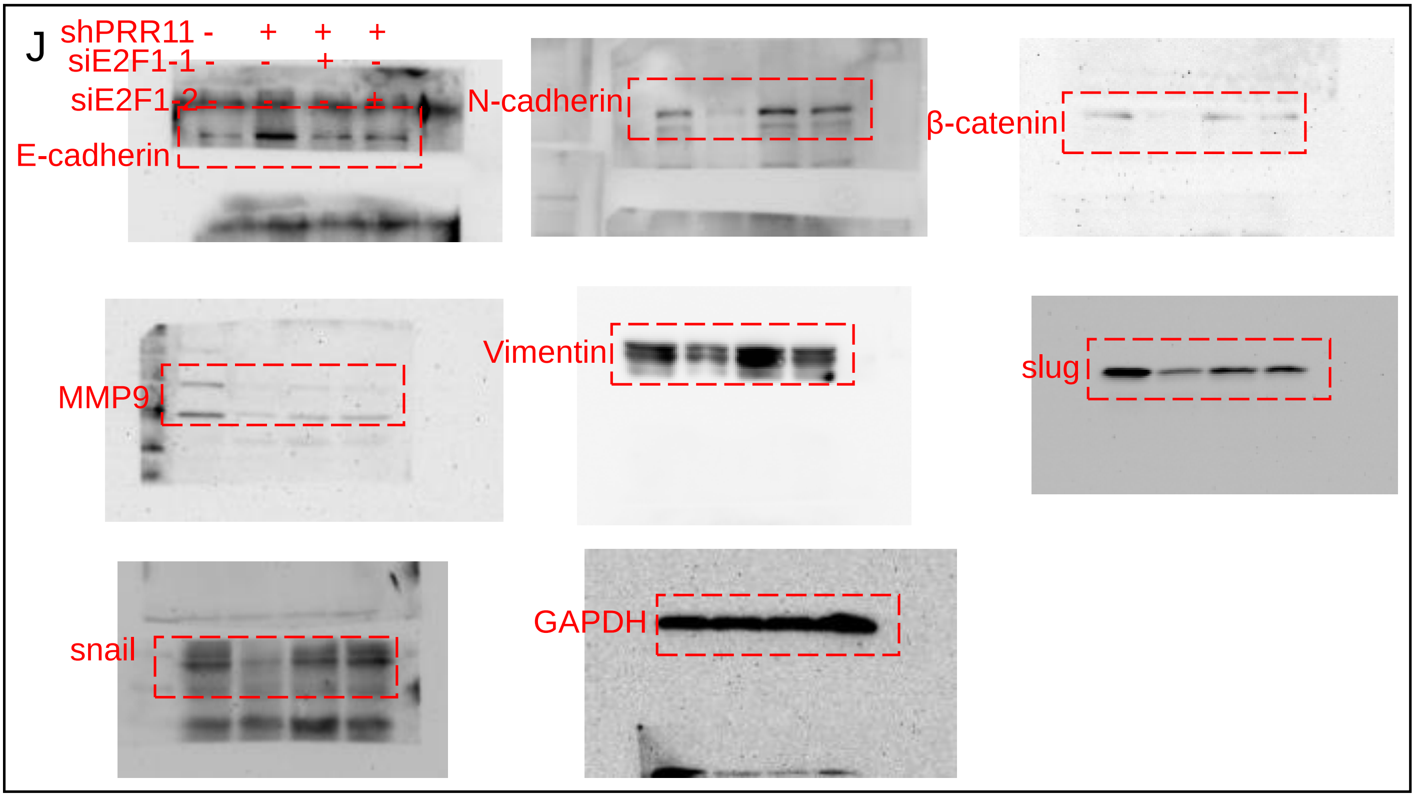

Full unedited gel for Supplementary Figure 5

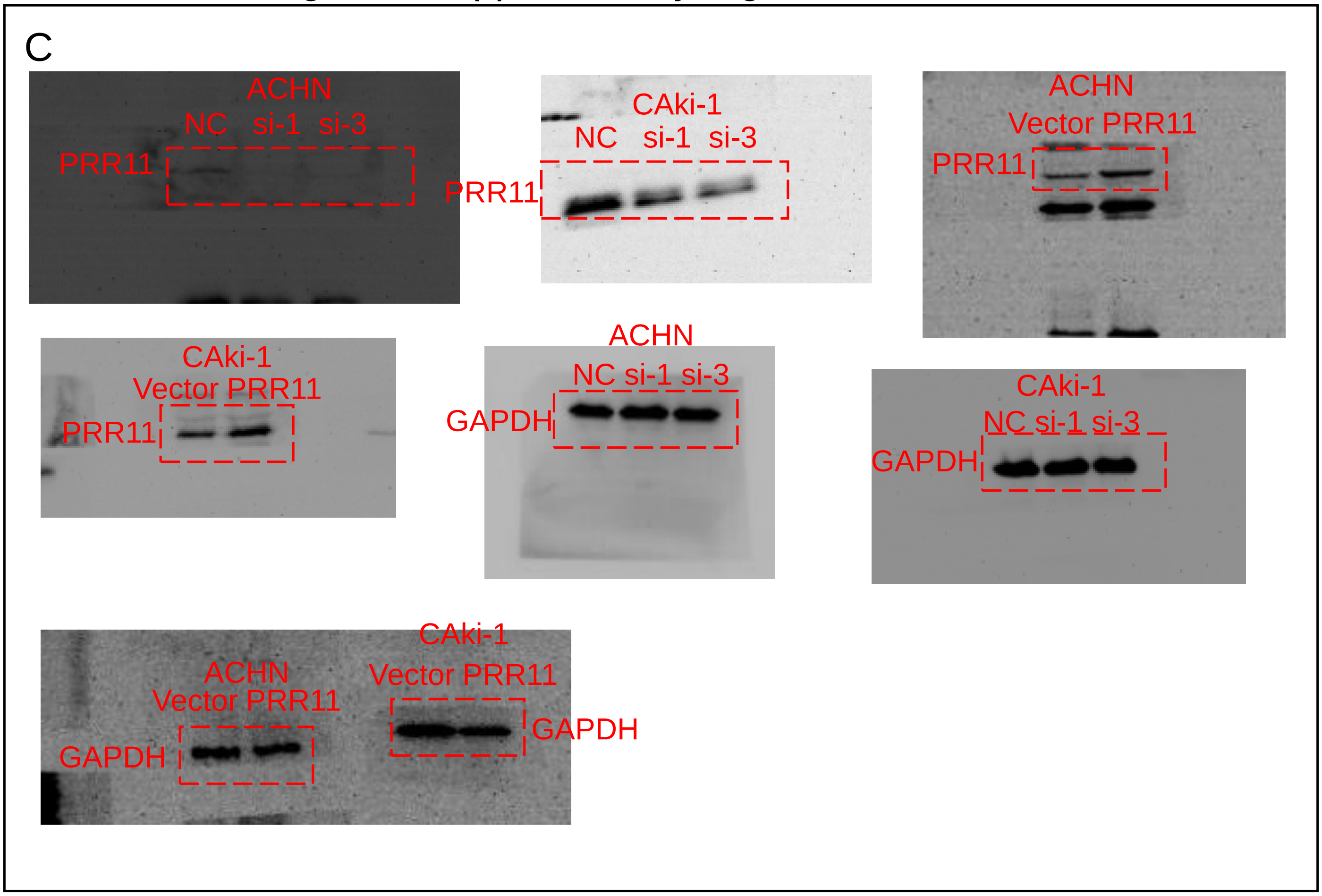

Full unedited gel for Supplementary Figure 7

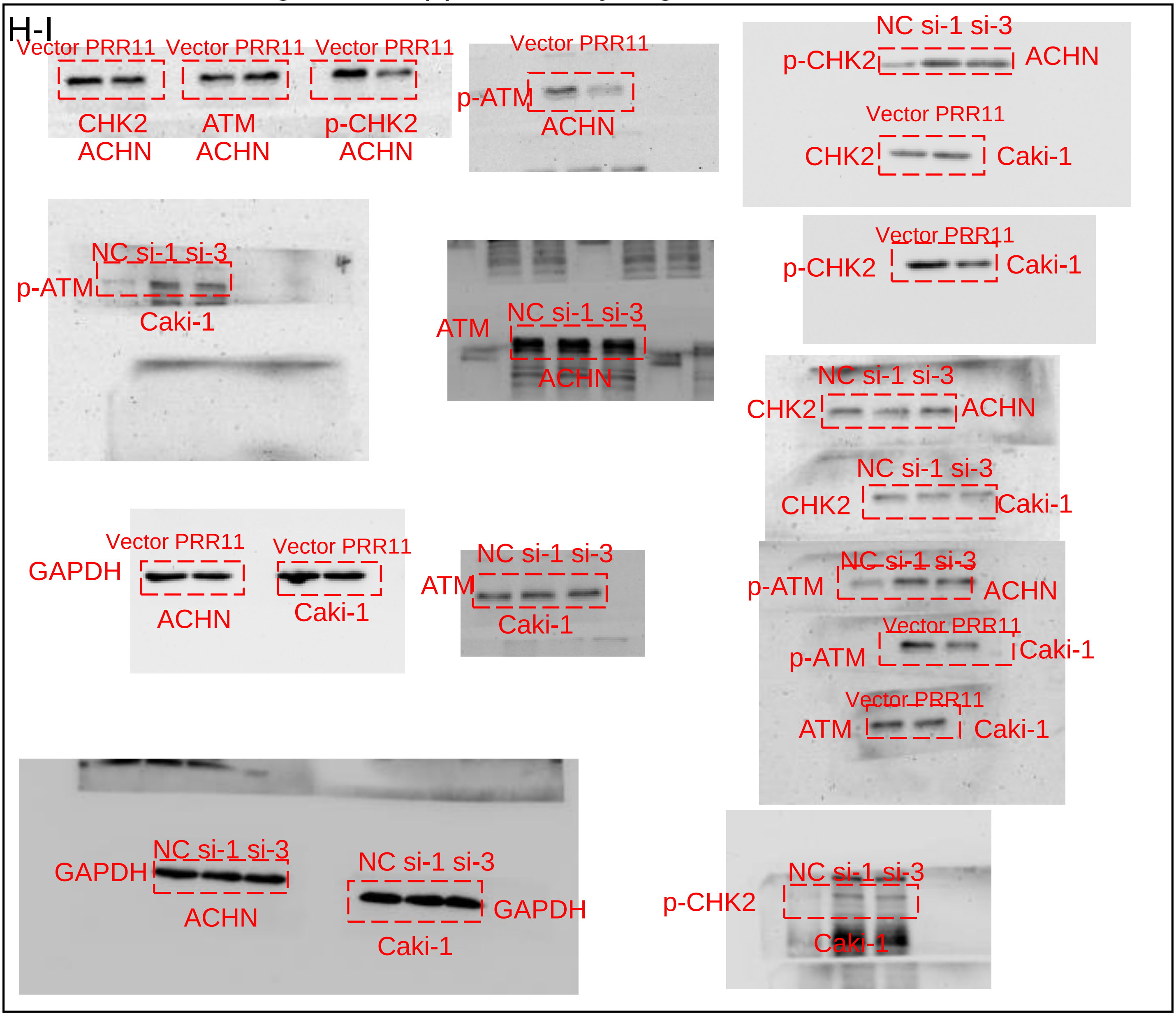

Full unedited gel for Supplementary Figure 8

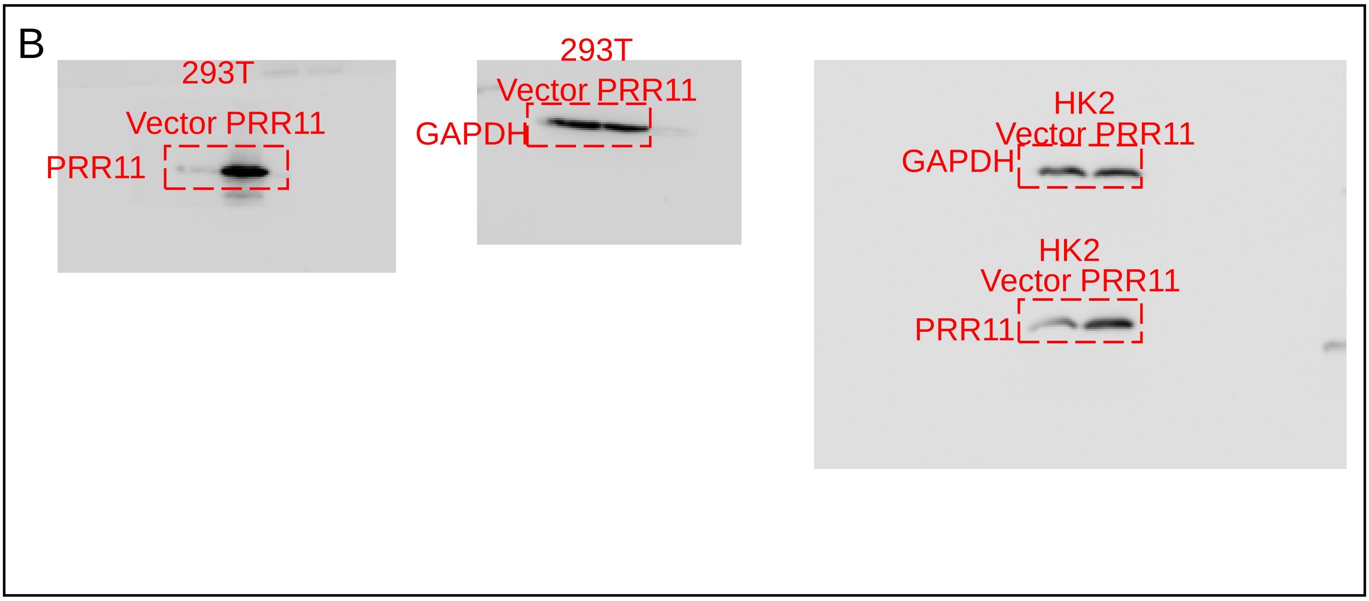

Full unedited gel for Supplementary Figure 11

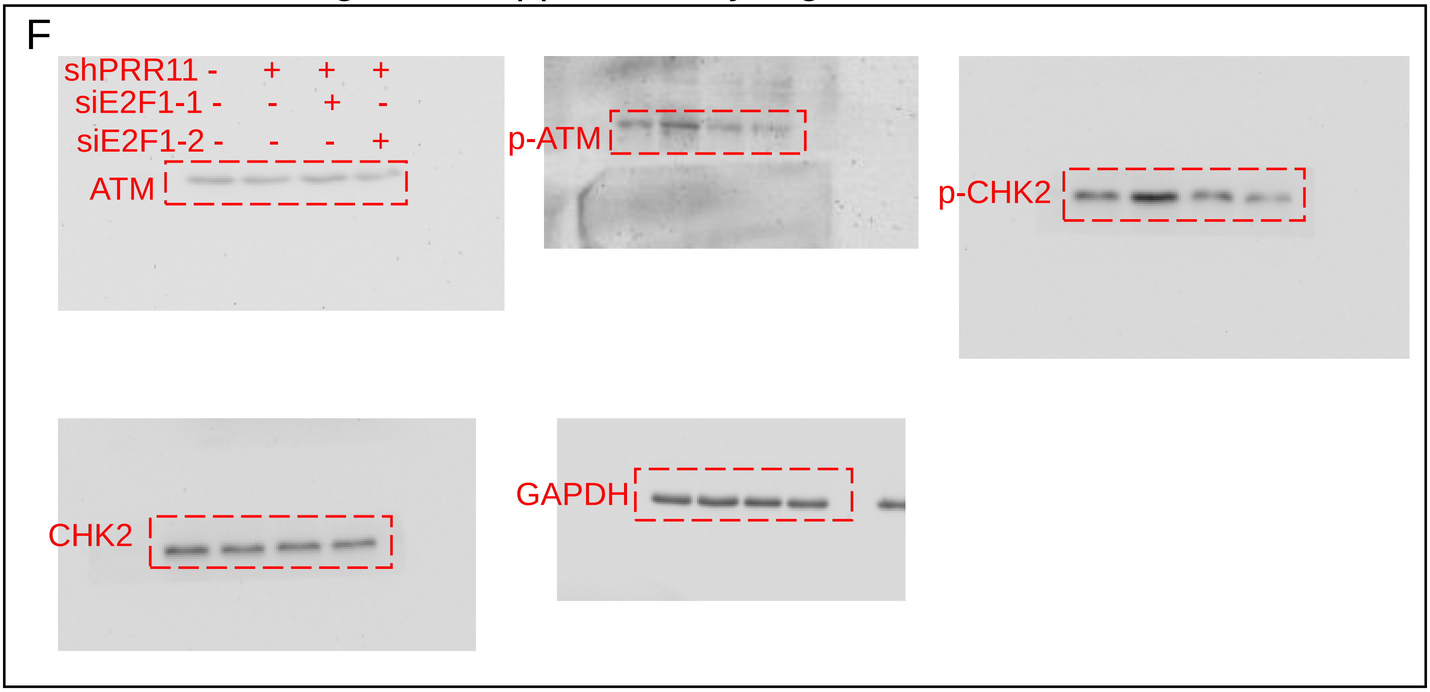

Supplement: Supplemental data [file jciinsight-6-145172-s315.pdf]
